# In Situ Insights into Enhanced Cooperative Ligand Exchange Kinetics via Solvent-Induced Restacking in a 2D Metal–Organic Framework

**DOI:** 10.1021/jacs.5c22455

**Published:** 2026-03-30

**Authors:** Richard Engemann, Irena Senkovska, Friedrich Schwotzer, Josefine Winkler, Volodymyr Bon, Susanne Machill, Philipp Wollmann, Fanny Reichmayr, Jonas Weiß, Johannes Scheffler, Ekin Esme Bas, Filip Formalik, Randall Q. Snurr, Dorothea Golze, Inez M. Weidinger, Eike Brunner, Stefan Kaskel

**Affiliations:** † Chair of Inorganic Chemistry I, 9169 Technische Universität Dresden, 01069 Dresden, Germany; ‡ Chair of Bioanalytical Chemistry, Technische Universität Dresden, 01069 Dresden, Germany; § Chair of Electrochemistry, Technische Universität Dresden, 01069 Dresden, Germany; ∥ Chair of Theoretical Chemistry, Technische Universität Dresden, 01069 Dresden, Germany; ⊥ Department of Chemical & Biological Engineering, 3270 Northwestern University, 2145 Sheridan Road, Evanston, Illinois 60208, United States; # Department of Micro, Nano, and Bioprocess Technology, 49567 Wroclaw University of Science and Technology, 50-307 Wroclaw, Poland

## Abstract

Understanding the reaction kinetics at catalytically active sites is crucial for integrating catalytic two-dimensional (2D) materials into industrial processes. This study focuses on *in situ* observation of ligand exchange kinetics and solvent-assisted structural restacking transition in the 2D paddle wheel-based MOF [Cu_2_(dttc)_2_]*_n_* (DUT-134­(Cu), dttc = dithieno­[3,2-b:2′,3′-*d*]­thiophene-2,6-dicarboxylate). The ligand exchange process, involving the replacement of dimethylformamide (DMF) with nitriles such as acetonitrile (ACN), pentanenitrile, and heptanenitrile, was investigated using advanced *in situ* characterization techniques with high temporal resolution, including powder X-ray diffraction and Raman spectroscopy. The larger analytes exhibited reduced exchange rates, consistent with enhanced steric hindrance and greater diffusion constraints. Interestingly, the study revealed that the exchange of DMF with ACN induces a structural transition to higher symmetry within few seconds, a transition from AB to AA stacking mode of the layers, and a widening of the interlayer distance. Crucially, this structural transition dramatically accelerates the solvent exchange process through cooperative effects, offering critical advantages for catalytic applications. Notably, the reverse exchange from ACN to DMF proceeds more slowly and does not reverse the structural changes, but a new phase is formed with preserved AA stacking. By isotope labeling of linker molecules in combination with two complementary theoretical vibrational simulation methods, the precise assignment of Raman bands and the vibrational modes associated with the ligand exchange process could be achieved. These pioneering insights into the dynamic behavior of 2D MOFs, coupled with ligand exchange, establish a highly promising and transformative approach to achieving enhanced tunability and responsiveness in future catalytic applications.

## Graphical abstract

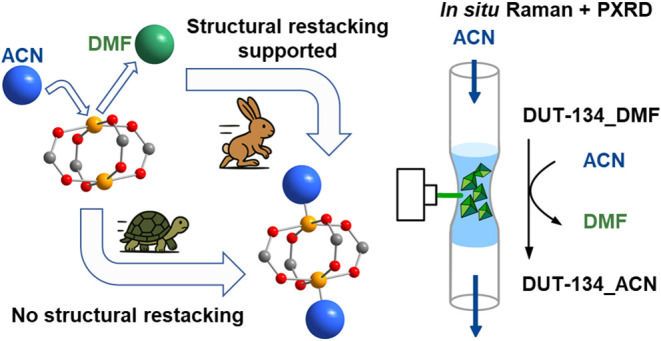

## Introduction

Metal–organic frameworks (MOFs) are an ideal platform for developing innovative concepts in science.^1^ Layered MOFs (2D MOFs) have emerged as a promising alternative to 3D frameworks due to their enhanced network flexibility and adaptivity, driven by the lability of layers, the diversity of layer stacking modes, and varying interlayer distance.^2–5^ This structural diversity is driven by interactions between layers involving also guest molecules, which can be controlled by the shape and chemistry of the guest. As a result, the 2D layers are susceptible to either in-plane (sliding) or out-of-plane motion, resulting in significant structural changes.^6–8^ In most cases, these transformations lead to beneficial properties, such as pore size adjustment and metal sites rearrangement.^9,10^ The structural flexibility of MOFs is a unique feature compared to other state-of-the-art porous materials, such as zeolites and activated carbons.^11–13^ Structural transitions in MOFs are often seen as a way to create responsive materials that can rapidly and reversibly adapt their structures and confinement, and thus a range of physical properties, in response to specific stimuli. This is an important feature for potential applications in gas storage and separation,^14–17^ sensing,^18–20^ artificial muscles,^21,22^ soft robotics,^23–25^ smart switches,^26–28^ and as metamaterials.^29–31^ An emerging field is that of adaptive MOF-based catalysts,^32,33^ where studies remain limited due to the complex interplay of factors influencing catalytic performance.

The M_2_(O_2_CR)_4_ paddle wheel unit is among the most prominent motifs in 2D MOFs, facilitating the formation of layers upon self-assembly with various dicarboxylic ligands.^34,35^ Already in 1998, one of the first representatives, [Zn_2_(bdc)_2_(H_2_O)_2_]*_n_* (MOF-2, bdc^2–^ = 1,4-benzene dicarboxylate), was reported by Yaghi and co-workers.^36^ The dynamics of prenucleation for paddle wheel clusters were later analyzed in detail by Nakamura et al.^37^ In 2001, Mori et al. reported a [Cu_2_(bdc)_2_]*_n_* material synthesized by combining Cu^2+^ ions with the same organic linker, which in its desolvated form adsorbs various gases such as nitrogen, argon, oxygen, and xenon.^38^ In 2009, Tannenbaum and co-workers reported on the structural transition of [Cu_2_(bdc)_2_]*_n_* upon desolvation^39^ and in 2014, the subsequent structural rearrangement was elucidated by the same group.^40^ Later on, a family of 2D structures based on paddle wheel (PWs) was reported, including a wide variety of dicarboxylate linkers.^41–43^ The PW typically exposes terminal axial sites, which are usually coordinated by the solvent used in the synthesis. These terminal ligands can be readily replaced by other ligands (or substrate molecules) in ligand-exchange reactions due to the weak metal–solvent interactions at these sites. Octahedral Cu^2+^ complexes are generally highly labile as a result of the Jahn–Teller distortion from ideal geometry, which leads to elongated bonds and weakly coordinated axial ligands.

Terminal ligand exchange in PW-based MOFs has been frequently reported to facilitate efficient desolvation and has been the subject of previous investigations. For example, by strategically exchanging different coordinating ligand molecules on the PWs of HKUST-1, the research group led by Zhu was able to enhance the accessible surface area of this material.^44^ Jeong et al. investigated the coordination of methylene chloride to the open metal sites on the paddle wheels in HKUST-1 and [Cu_2_(bdc)_2_]*_n_*. By analyzing the Raman active Cu–Cu vibration, the formation of coordination bonds was monitored.^45^ The kinetics of ligand exchange were studied by Matzger et al.^46^ However, such studies remain rare, particularly in the context of 2D MOFs, where they have yet to be explored. 2D paddle wheel (PW) based MOFs offer accessible metal sites as potential catalytic centers.^47–49^ The Lewis-acidic metal sites can be employed for various important reactions, such as epoxide hydroxylation^50^ or carbohydrate conversion.^51^ In order to design efficient catalysts, it is important to consider the kinetics of substrate binding and desorption from the active site, as these are often found to be the rate-limiting steps in the overall catalytic process.^52,53^

[Cu_2_(dttc)_2_]*_n_* (DUT-134), as a 2D MOF representative, is consists of Cu_2_–PWs connected by a bent dithieno­[3,2-b:2′,3′-*d*]­thiophene-2,6-dicarboxylate (dttc) as linker into a (4,4) net.^54^ The compound can be assembled into large-scale superstructures through a bottom-up approach.^55^ A unique feature of this material is its guest-dependent exfoliation, resulting in nanoscale sheets, and guest-dependent framework flexibility.^54^ It has been demonstrated that the coordination of organic solvent molecules to the axial PW positions influences the interlayer spacing in the crystal structure. Ketones appear to result in the largest interlayer distance, whereas similarly sized alcohols constrict the layers, with the layer separation approaching that of the desolvated structure.

In the following, we investigate the kinetics of terminal ligand exchange using advanced complementary *in situ* techniques: Raman spectroscopy and powder X-ray diffraction. As substrates for the kinetic study, nitriles were chosen, since they are employed in several important organic reactions, such as hydrogenation to primary amines^56–58^ or oxidation to amides.^59,60^ Fast *in situ* Raman spectroscopy enables real-time monitoring of local coordination environments, with characteristic signal changes directly revealing exchange rates. *In situ* powder X-ray diffraction gives valuable insights into the collective structural rearrangements, including layer restacking and symmetry changes. To unambiguously assign the experimentally observed vibrations in Raman spectra to the respective groups, two theoretical simulation methods were used. Experimentally, the ^13^C- and ^18^O-labeling of specific linker groups was vital to confirm the calculation results. Understanding ligand exchange kinetics coupled to structural transformations provides important insights into intrinsic and defect active sites and can be considered an essential analytical technique for precision chemistry of 2D materials in the future.

## Results and Discussion

### Crystal Structure of DUT-134

In the structure of as-made [Cu_2_(dttc)_2_(DMF)_2_]*_n_* (DUT-134, denoted as **2**), the Cu-containing paddle wheel units are connected by dttc linkers into a layer with (4,4) net topology (Figure 1a).

**Figure 1:**
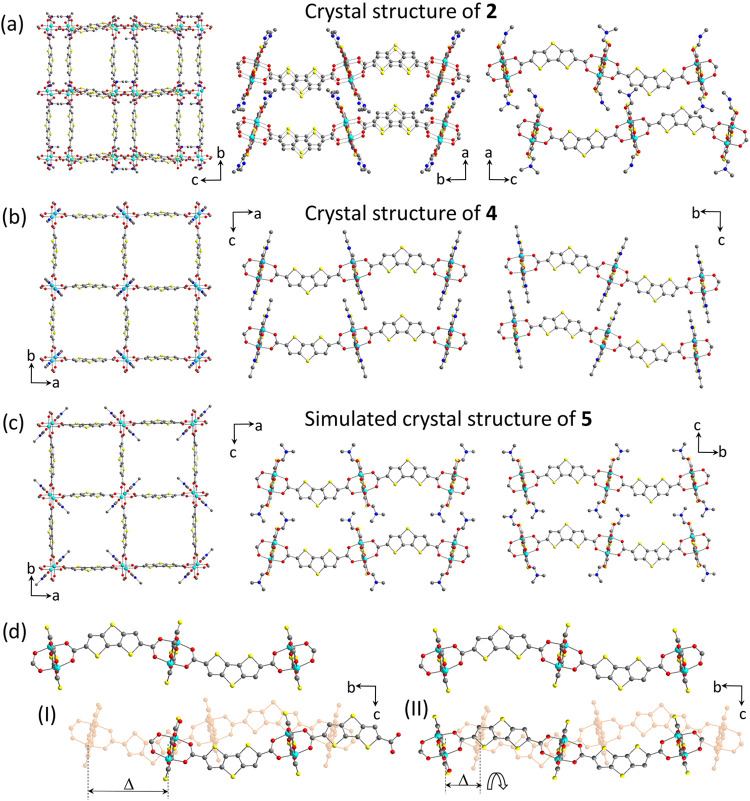
View on the crystal structure of **2** (a), **4** (b), and **5** (c) along corresponding crystallographic axes; (d) possible **2** to **4** transition pathways. The layer in light orange corresponds to the initial position of the second unit cell layer in **2**. Left: in-plane shift of the layer (17.0 Å) to reach the position in **4**; right: in-plane shift (4.5 Å) and 180° turn to reach the position in **4**.

The layers extend along the (011) plane, whereby each unit cell contains two symmetry-related layers, and the structure features square-shaped pore channels running along the *a*-axis. The pore limiting diameter is 7.1 Å, according to the pore analyzer tool implemented in the Mercury software.^61^ There are two types of solvent molecules present in the solvated MOF: those coordinated to the Cu centers, as well as those disordered, uncoordinated inside the pores. The layers in the DMF-containing structure **2** are separated by 8.9 Å (*a* = 17.880 Å, two layers/unit cell), a distance dictated by the coordinated DMF molecules. As reported earlier,^54^ soaking of **2** in different organic solvents leads to the exchange of solvent in the pores, as well as the displacement of the coordinated species on the terminal positions of the PW. As a result, a structural transition leading to a change in the interlayer distance is initiated. Alcohols, such as ethanol, reduce the interlayer distance from 8.9 Å to 7.1 Å.

However, complete desolvation of the structure (removal of coordinated and uncoordinated solvent) through supercritical CO_2_ drying results in a further decrease in the interlayer distance to merely 6.1 Å. In the present study, the DMF exchange by nitriles, such as aceto-, penta-, and heptanenitrile, was studied (Figure 2).

**Figure 2:**
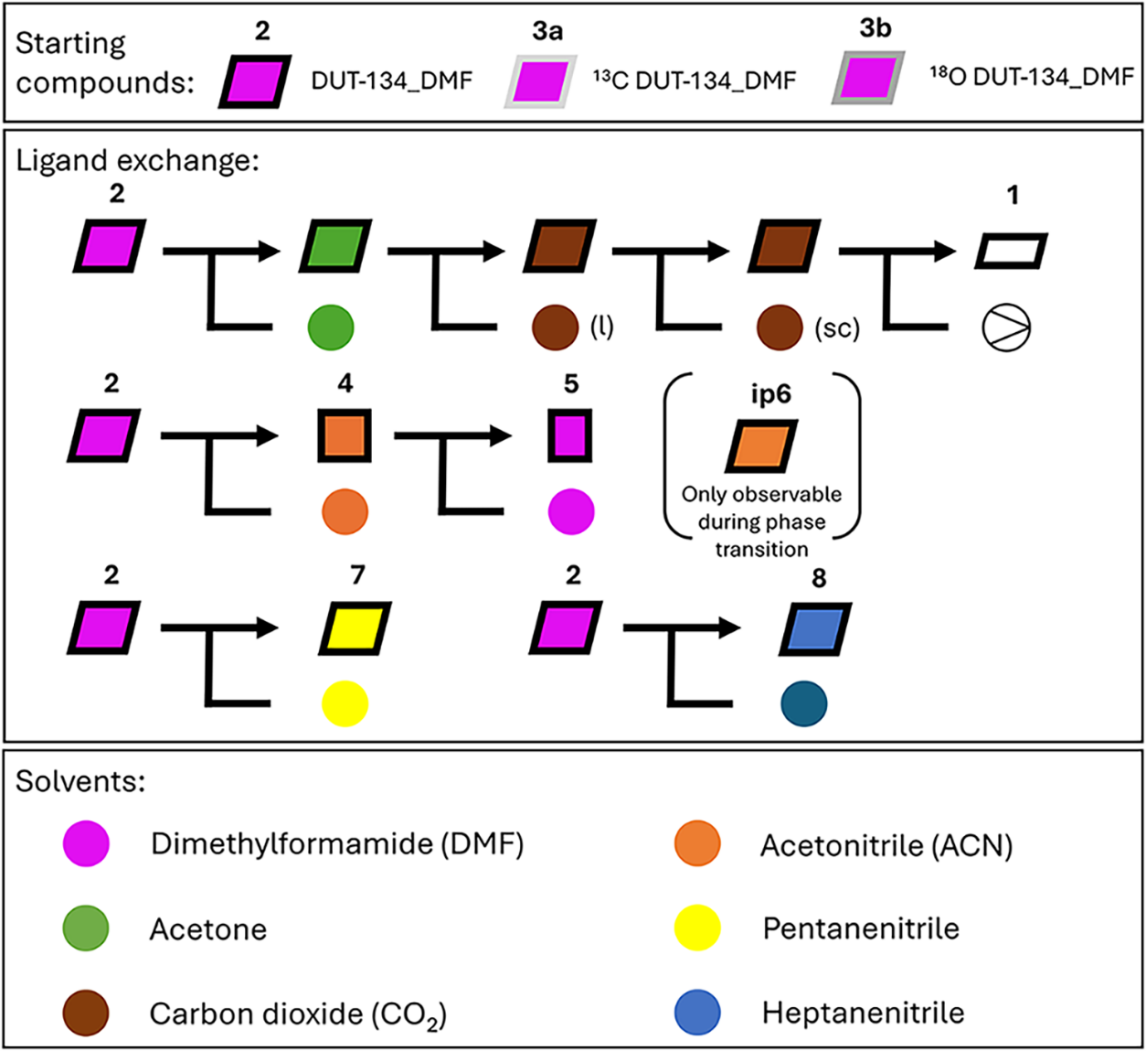
Overview of the compounds and ligand exchange processes discussed herein. The main starting compound for the ligand exchange is [Cu_2_(dttc)_2_(DMF)_2_]*_n_* (DUT-134_DMF), further denoted as **2**. **3a** and **3b** are DUT-134_DMF compounds, where the carboxylate carbon (**3a**) or oxygen (**3b**) atoms were isotope-labeled with ^13^C and ^18^O during the synthesis (see SI, Sections 5 and 6). The structural transition from MOF containing DMF (**2**) to the solvent-free structure (**1**) proceeds upon supercritical CO_2_ drying, where the DMF ligands (purple) coordinating the Cu-centers are exchanged to acetone (green), liquid CO_2_ (brown, l), and supercritical CO_2_ (brown, sc). The ligand exchange in **2** with acetonitrile (orange) yields compound **4**, whereas the exchange back to DMF yields compound **5**. The formation of an intermediate phase (**ip6**) during structural transition from **2** to **4** is only observable for a short time by *in situ* PXRD. The DMF exchange in **2** to pentanenitrile (yellow) yields compound **7**, while the exchange with heptanenitrile (blue) yields compound **8**. The diamond-shaped symbols represent structures with AB stacking, while rectangles indicate structures with AA stacking. Differences in width and height indicate a change in layer spacing.

### Exchange of DMF with Nitriles

Crystals of **2** were synthesized according to the procedure described by Schwotzer et al.^54^ (see SI, Section 5). The phase purity and structural integrity were proven by PXRD. To exchange the DMF ligands with nitriles, crystals of **2** were immersed in acetonitrile over several days to yield DUT-134_ACN (**4**). The comparison of the PXRD patterns of samples before and after solvent exchange (see SI, Section 7) reveals an unexpectedly pronounced change in the diffraction pattern.

Previous solvent exchange experiments,^54^ in which DMF was replaced by alcohols, ketones, or DMSO, resulted only in a shift of the characteristic 200 peak, indicative of a change in the interlayer distance.

In the case of **4**, however, the PXRD pattern significantly differs from that of **2**. Therefore, **4** was subjected to single-crystal X-ray diffraction to confirm the structural transformation. The crystal structure solution indicates an increase in the space group symmetry, namely, the space group changes from *Pnma* (group number 62) in **2** to *P*4/*nmm* (group number 129) in **4** upon ligand exchange. *Pnma* is an indirect subgroup of *P*4/*nmm* with a most probable pathway of *Pnma*–*Pnmm*–*P*4/*nmm*, previously observed, for example, in BiTeX (X = I, Br) compounds subjected to high pressures.^62^ While the in-plane distances remain almost unchanged (*b* = 27.930 Å, *c =* 27.850 Å in **2**; *a = b* = 28.090 Å in **4**), a substantial increase in the interlayer distance is evident (Figure 1a,b). The lattice parameter corresponding to the stacking direction increases from 17.880 Å (*a* in **2**) to 20.300 Å (2 x *c* in **4**). The transition implicates a change of the layer stacking mode from an AB-type in **2** to an AA-type in **4** (Figure 1a,b).

The pore limiting diameter increases to 10.6 Å in **4** (compared with 7.1 Å **2**). After a detailed analysis and comparison of the crystal structures of **2** and **4**, it is evident that such a structural transformation can potentially proceed in two different manners (Figure 1d): (I) A shift of the individual layers with respect to each other along the *b*-axis to match the AA-stacking of **4** (17.0 Å); (II) A much smaller shift in *b* direction (4.5 Å), accompanied by a 180° rotation of the layer (which is an unlikely scenario).

In the following experiment, the DUT-134_ACN (**4**) crystals, which were obtained through a postsynthetic ligand exchange approach, were re-exposed to DMF. However, a detailed analysis of the PXRD data, and Pawley refinement combined with simulations (for more details see Section 14 of SI) revealed that this process did not result in a structural transformation from **4** back to **2**. Instead, the crystal structure remained in the *P*4/*nmm* space group, resulting in a new structure designated as **5** (Figure 1c). A negligible increase in the in-plane lattice parameters from *a* = *b* = 28.090 Å in **4** to 28.113 Å in **5** is accompanied by an expansion in the interlayer distance from 10.150 Å (in **4**) to 10.666 Å (in **5**). In this case, it is evident that the MOF prefers the accommodation of introduced DMF in the ligand exchange process, preserving the AA stacking of the layers. The hysteresis in the solvent exchange process potentially opens up the possibility of comparing the kinetics of interlayer distance expansion and more complex structural transitions, both coupled with the solvent exchange.

Exchange of DMF in **2** with pentanenitrile (giving compound **7**) and heptanenitrile (resulting in compound **8**) leads to changes in the interlayer distances only and does not lead to changes in the stacking mode, according to PXRD analysis (see SI, Sections 7 and 14).

### Experimental Raman Spectra and Band Assignment Strategies

Characteristic differences between the MOF structures discussed earlier were identified in Raman spectra collected for the crystals of **2**, **4**, **5**, **7**, and **8** (see SI, Section 12). Typically, the vibrations of the organic linker are predominantly observed within the range of 500–1800 cm^–1^, while the modes associated with metal–ligand bonds or lattice vibrations appear at frequencies below 500 cm^–1^.^63^ Theoretical simulations and isotope labeling of the linker was pursued to ultimately assign the experimental bands in the Raman spectra of DUT-134 to the vibrational modes of the MOF structure. The latter causes a characteristic isotopic shift of metal–ligand vibration if the α-atom of the ligand (atom directly bonded to the metal) is substituted.^64,65^

### Calculations of Raman Vibrational Modes for Desolvated DUT-134 (**1**)

To gain initial insight into the vibrational spectra of DUT-134, phonon simulations were performed for the solvent-free DUT-134 crystal structure **1**.^54^ The data calculated using two different approaches (see Experimental Section) revealed a comprehensive ensemble of Raman vibrations and their associated wavenumbers (see SI, Section 11). Finally, the calculated Raman spectra, including intensity of the peaks, were obtained at the density functional theory (DFT) level using normal-mode analysis, which were in good agreement with one observed experimentally (Figure S17).

In the next step, calculations were performed using the crystal structure of compound **2** as a starting point. The comparison of experimental and theoretical spectra is shown in Figure 3.

**Figure 3:**
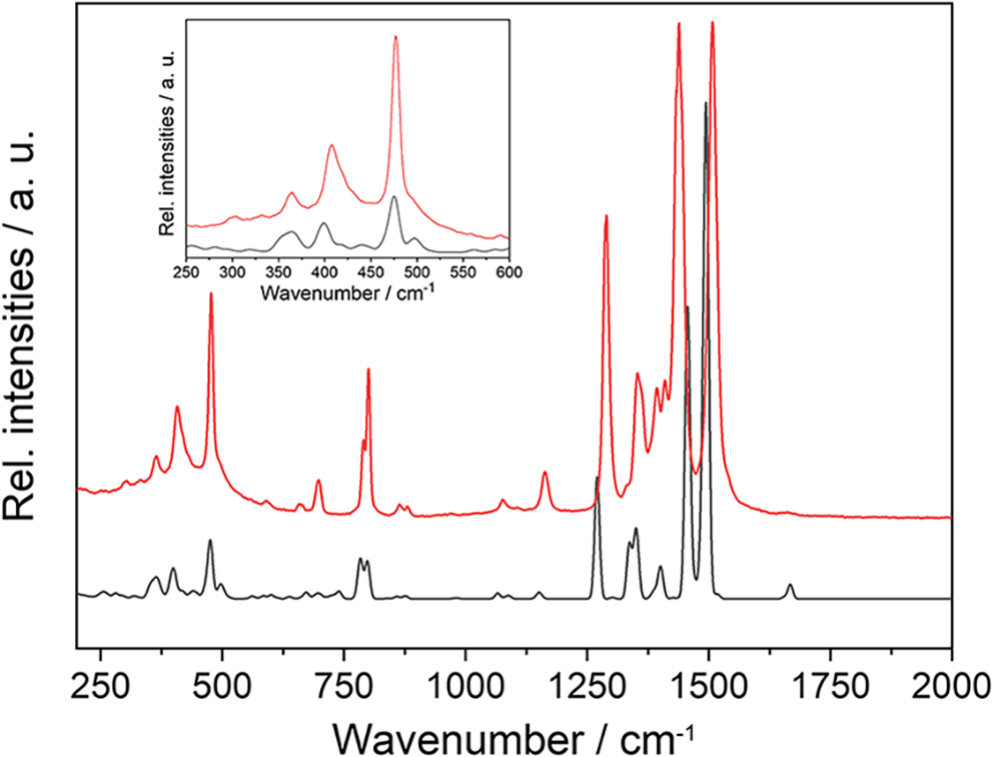
Experimental (red) and calculated (black) Raman spectrum of **2**.

Thus, the Raman bands observed experimentally in Raman spectra of **2** could be assigned to the vibrational modes of the MOF structure (Table 1). The bands at 367 cm^–1^ and 408 cm^–1^ originate from vibrations involving the paddle wheel and coordinated solvent, which would therefore be suitable to study solvent exchange. The Raman vibrations of the carboxylic group are also visible at about 800 cm^–1^, with dominant in-plane vibrations at 792 cm^–1^ and 804 cm^–1^ being particularly noteworthy. As expected, vibrations associated with the dttc skeleton are observed in the 1200 cm^–1^ to 1500 cm^–1^ region. The symmetric and asymmetric valence vibrations at 1408 cm^–1^ and 1507 cm^–1^ belong to carboxylic groups. A visual representation of the herein discussed vibrations based on the performed calculations can be accessed through a Zenodo repository.^66^ The unambiguous assignment of these vibrations, however, was achieved through experiments on isotope labeled compounds (Section 10 of SI).

**Table 1:** Vibrations and Associated Structural Motifs of **1** and **2** Assigned on the Basis of the Performed Calculations^a^

type of vibration	wavenumber calc. for **1** (cm^–1^)	wavenumber calc. for **2** (cm^–1^)	wavenumber exp. of **2** (cm^–1^)	wavenumber exp. of **3a** (cm^–1^)	wavenumber exp. of **3b** (cm^–1^)
ν_as_(O=C–O); *δ(N–[CH]_3_) of DMF	1501	1514*	1507	1509	1504
ν_as_(OO[C–C=C]–H); ν(S–C–C–S)	1483	1494	1507	1509	1504
ν(OO[C–C=C]–C); ν(S–[C–C]–S); *δ(N–[CH_3_]) of DMF	1436	1457*	1437	1437	1437
ν_s_(O=C–O); ν(OO[C–C=C]–C); ν(S–[C–C]–S); *δ(N–[CH_3_]) of DMF	1397	1406*	1408	–	1378
collective dttc vibration: ν(OO[C–C=C]–C); ν(S–[C–C]–S)	1383	1399	1403	1406	1410
collective dttc vibration: ν(OO[C–C=C]–C); ν(S–[C–C]–S)	1331	1354	1351	1350	1349
collective dttc vibration: ν_s_([C=C])	1271	1271	1288	1289	1288
ν(OO[C–C=C]–H)	1150	1157	1161	1160	1156
ν(OO[C–C=C]–H)	1063	1064	1077	–	–
synchron in plane scissoring δ(O=C–O); ν(OO[C–C=C]–H)	787	799	804	798	781
asynchron in plane scissoring δ(O=C–O); ν(OO[C–C=C]–H)	773	784	792	791	776
synchron out of plane rocking γ(OO[C–C=C]–H)	740	744	786	787	766
in-plane stretching δ(C–S–C)	475	475	481	480	477
ν_s_(Cu–O); δ_as_(C–S–C); *δ(N–[CH_3_]) of DMF	402	410*	408	406	398
ν_as_(Cu–O); rocking vibration ρ(dttc); *δ(N–[CH_3_]) of DMF	365	367*	367	364	354

a Experimentally obtained data of **2**, **3a**, and **3b** are also given. An in-depth discussion of the experimental results for **1** can be found in Section 10 of SI.

### Raman Spectroscopy on Isotope-Labeled DUT-134

The H_2_dttc linker, containing ^13^C- or ^18^O-labeled atoms, was synthesized by carboxylation of 2,6-dibromodithieno­[3,2-b:2′,3′-*d*]­thiophene with ^13^C- and ^18^O-labeled CO_2_, respectively (for more details see SI, Sections 2–4). The linkers obtained were utilized for the synthesis of MOFs (Section 6, SI), to form the isotope-labeled DUT-134 variants ^13^C-DUT-134 (**3a**) and ^18^O–DUT-134 (**3b**). The substantial agreement of theoretical and experimental powder X-ray diffraction patterns indicates the anticipated crystal structure and phase-pure products (Figure S5). Raman spectra collected for **3a** and **3b** indeed reveal shifts in the positions of some peaks in comparison to **2** (Figure 4b). The shift is particularly evident for Raman bands at 367, 408, 481,793, 804, 1288, 1351, 1392, 1408, and 1507 cm^–1^ observed for **2** (Figure 4). Notably, significant shifts over 10 cm^–1^ could be observed for 367, 408, 793, 804, 1392, and 1408 cm^–1^, in the spectrum of **3b**. The wavenumber observed in Raman spectroscopy depends on the reduced mass of the vibrating system, showing an inverse relationship. Accordingly, vibrational modes of MOFs involving ^13^C- and ^18^O-labeled atoms are expected to appear at lower wavenumbers compared to the unlabeled reference compound. Raman measurements confirmed this trend, and it can be assumed that all of these Raman bands involve vibrations in the vicinity of the isotope-labeled carboxylate group, including the vibration of the group itself, as well as some vibrations of the dttc core (Table 1). These findings are consistent with theoretical calculations.

**Figure 4:**
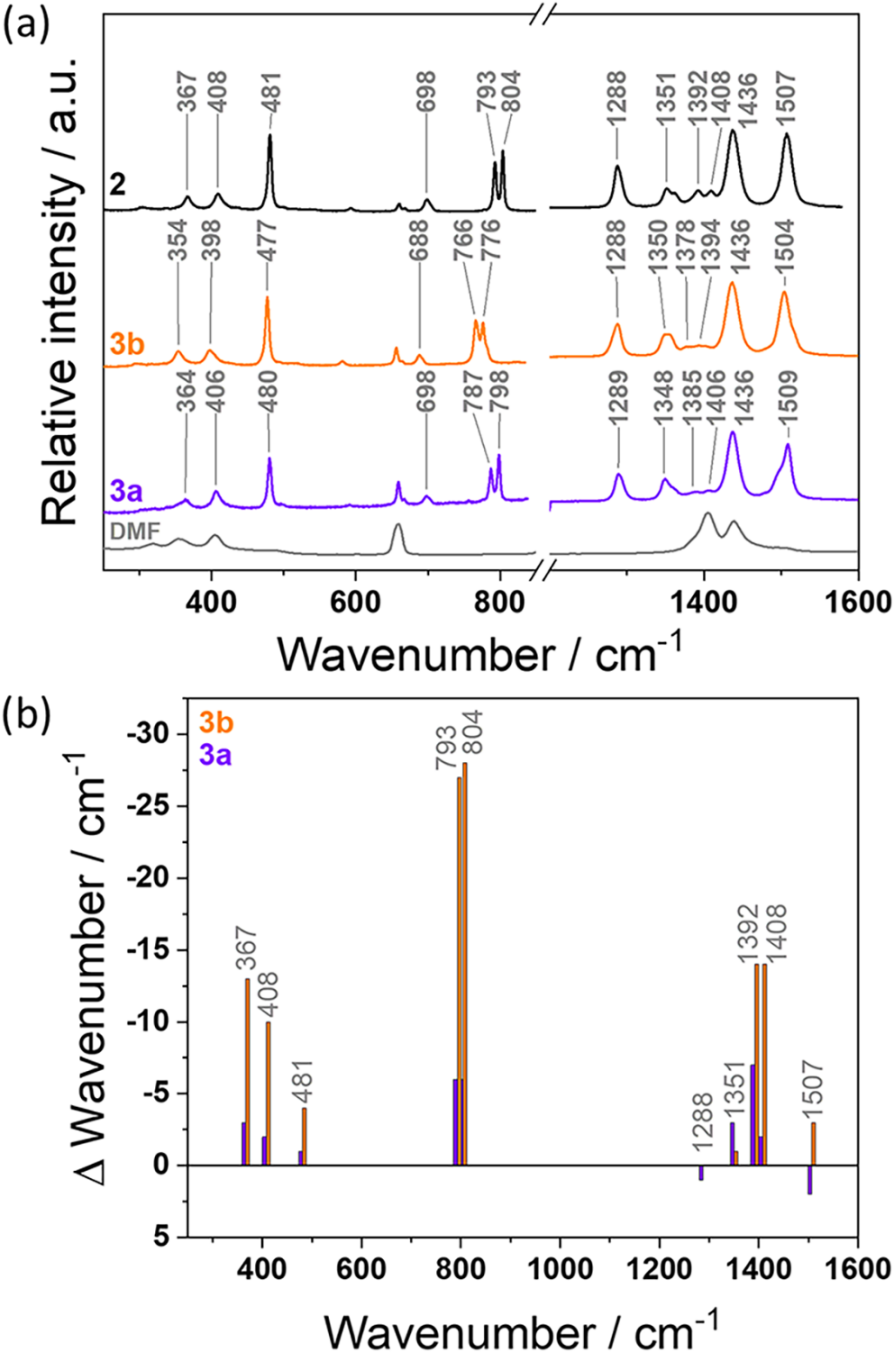
(a) Comparison of Raman spectra of nonisotope-labeled **2** (black), ^13^C-isotope-labeled **3a** (purple), ^18^O-isotope-labeled **3b** (orange), and DMF (gray). (b) Peak shifts extracted from Raman spectra are shown in purple for **3a** and in orange for **3b**, with the peak positions in nonisotope-labeled **2** serving as the zero line. The wavenumber differences are given above.

Building on the Raman band assignments and the structural transition observed during the exchange from DMF to ACN, the kinetics of the solvent exchange process were investigated using two complementary techniques: *in situ* Raman spectroscopy and *in situ* PXRD. For *in situ* Raman spectroscopy measurements, it would be most effective to monitor the shift in the Cu–O stretching vibration, which appears at approximately 408 cm^–1^ in **2** and **5**, while in **4**, it is approximately at 411 cm^–1^, confirming the effectiveness of this band for monitoring solvent exchange on the paddle wheel. The shift to larger wavenumbers indicates the strengthening of the Cu–O bond and suggests a weaker interaction between ACN and the copper centers in comparison to DMF.

### *In Situ* Raman Spectroscopy

The kinetics of solvent exchange were monitored using *in situ* Raman spectroscopy, collecting data from both the solid phase and the surrounding liquid phase. Crystals of DUT-134_DMF (**2**) were placed and fixed in a glass capillary (Figure 5a).

**Figure 5:**
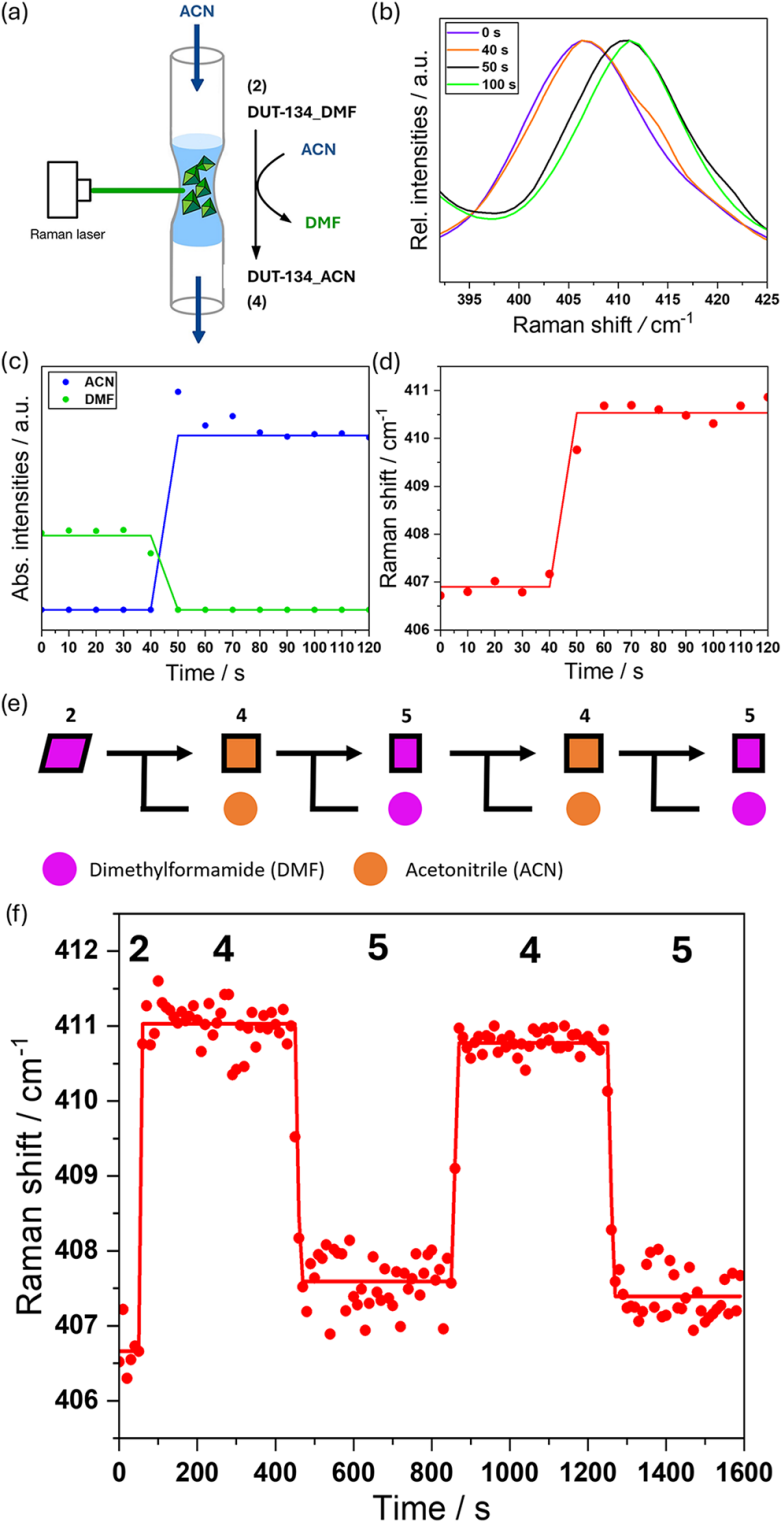
Solvent exchange kinetics in DMF-containing DUT-134 (**2**). (a) Schematic illustration of the *in situ* Raman experiment during the exchange of DMF with ACN from **2** to **4**. (b) Raman peak shift of the Cu–O stretching band during the solvent exchange. (c) Absolute Raman band intensities of the bands at 860 cm^–1^ (DMF, green) and at 2251 cm^–1^ (ACN, blue) as a function of solvent exchange time. (d) Wavenumber change of the Cu–O stretching Raman band during solvent exchange. (e) Schematic illustration of the MOF state upon cycling solvent exchange. Starting from **2**, containing DMF (purple), which was exchanged with ACN (orange) to give **4**. The reverse exchange of ACN with DMF leads to **5**. (f) Wavenumber shifts of the Cu–O stretching Raman band during the cycling solvent exchange of DMF and ACN.

The solvent (ACN) flow was injected using a syringe pump, and Raman spectra were acquired at 10 s intervals. The laser was focused on the crystal’s surface. Experimental fluctuations, particularly those related to band intensities, are probably attributed to the slight motions of the crystallites within the flow cell.

To experimentally determine the time at which ACN reaches the crystals (time zero for the solvent exchange), the intensities of the strong vibrational band at 860 cm^–1^ originating from N–CH_3_ stretching and characteristic for DMF, as well as at 2251 cm^–1^, characteristic for N≡C stretching of ACN, were monitored (Figure 5c). Thus, the N≡C stretching band of ACN at 2251 cm^–1^ emerges instantaneously after 40 s of the experiment, indicating that the ACN has reached the crystals. The signal intensity reaches a maximum after approximately 10 s and a plateau afterward. In parallel, the intensity of the N–CH_3_ stretching band of the initially present DMF at 860 cm^–1^ remains constant during the dead time of 40 s and rapidly decreases between 40 and 50 s until it reaches the detection limit, indicating that DMF in the capillary is entirely replaced by ACN also within approximately 10 s between 40 and 50 s of experiment time. The peak position of the Cu–O stretching band in the Raman spectra shifts during the replacement of the initially present coordinating DMF solvent by ACN (Figure 5b,d). The band shifts in the time between 40 and 50 s from 407 cm^–1^ to 411 cm^–1^, indicating that the binding energy between copper and oxygen increases upon ligand exchange. A further shift beyond 50 s is not observed, indicating that the exchange of solvent on copper centers and the structural transition associated with the initial exchange of DMF by ACN is completed within 10 s (Figure 5d).

This is notably much faster than the approximately 120 min reported for solvent exchange in [Cu_3_(btc)_2_]*_n_* single crystals, where DMF was replaced by EtOH.^46^ This discrepancy, in addition to differences in the crystal structure and topology, is evidently attributed to the experimental conditions and measurement setup. In the case of [Cu_3_(btc)_2_]*_n_*, the solution placed into the NMR tube above the crystals was monitored to track the increase in DMF concentration over time as DMF exited the framework, causing transport-limited kinetics controlled by the solvent diffusion within the tube. In contrast, the flow cell exhibits enhanced efficiency in terms of solvent exchange, dissipation, and diffusion.

The repeatability of solvent exchange was assessed in a cyclic experiment, in which **2** was alternately exposed to ACN and DMF in the flow cell setup (Figure 5e).

The dead time of the experiment was again monitored by observing the intensity changes of the N–CH_3_ stretching band of DMF at 860 cm^–1^ and the N≡C stretching band of ACN at 2251 cm^–1^ (Figure S27). Compound **5** exhibited an average Cu–O band position of 407.5 cm^–1^, whereas the Cu–O band in **4** consistently appeared at ca. 411 cm^–1^ (Figure 5f).

The difference in Cu–O band wavenumbers observed between **2** and **5** may be attributed to structural differences between the two compounds and to the limit of detection for the detector used. The data analysis revealed that the transformation from **2** to **4** occurs within a time frame of approximately or even less than 10 s, whereas the reverse transformation from **4** to **5** requires a longer time of 30 s, although the structural changes are less complex in this case. The second transformation from **5** to **4** is completed in about 20 s, which was also longer than the previously observed value of ≤ 10 s. Thus, it is logical to assume that the phase transition, being energetically preferable, accelerates the solvent exchange process due to a cooperative adsorption mechanism.^67–69^ Therefore, the kinetics of solid-state phase transition was studied *in situ* by powder X-ray diffraction (PXRD).

### *In Situ* Powder X-ray Diffraction

The exchange of DMF by ACN was monitored *in situ* by powder X-ray diffraction in the same flow cell setup using synchrotron radiation (PETRA III, Hamburg) in one-second intervals. To estimate the zero time, the changes in amorphous background were analyzed (for further details, see SI, Sections 15 and 16). The dead time for this setup configuration was determined to be 145 s (Region I, Figure 6a,b). Analysis of PXRD patterns following the introduction of ACN to crystals of **2** using a syringe pump revealed complex changes in the powder pattern (Figure 6a,b). Analysis of the PXRD patterns before ACN introduction results in a phase very similar to that known as DUT-134­(Cu)_DMF (*a* = 17.82 Å). In the first four seconds after ACN introduction, several reflections, *i*.*e*. the 120, 112, 200, and 202 reflections, involving the nonzero *h* index sensitive to the interlayer separation, experience a continuous shift to higher 2Θ values (Region II, Figure 6a,b).

**Figure 6:**
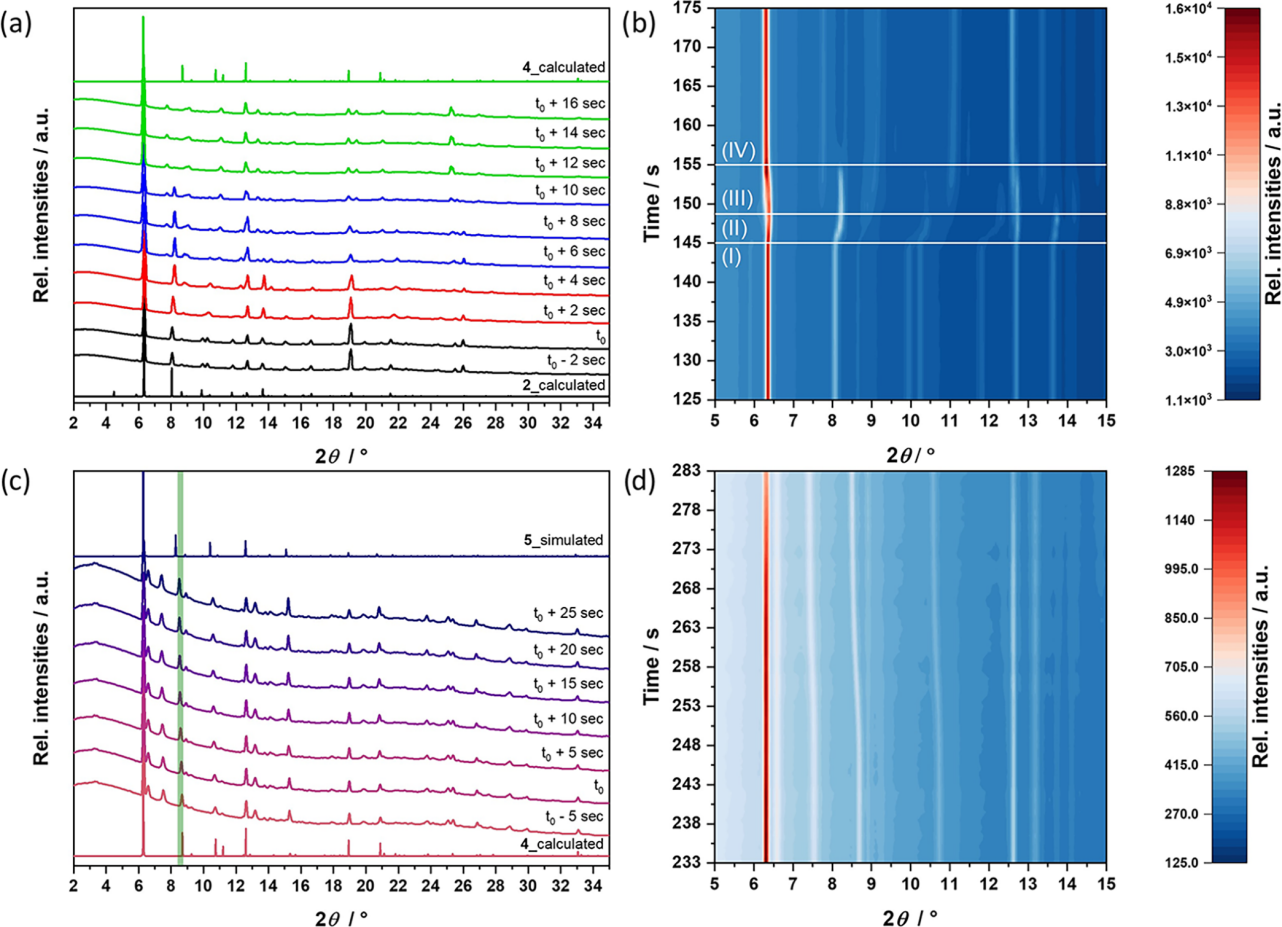
(a) *In situ* powder X-ray diffractograms collected upon exchange of DMF in **2** with ACN displayed in 2 s intervals. Diffractograms collected before the addition of ACN (Region I) are depicted in black, while the regions of phase transition are depicted in red (Region II), blue (Region III), and green (Region IV). (b) *In situ* powder X-ray diffractograms during the exchange of DMF in **2** by ACN, with the region of phase transition borders marked in white. (c) *In situ* powder X-ray diffractograms collected upon exchange of ACN in **4** by DMF (depicted in 5 s intervals). The 001 reflection shift over the exchange period is marked in green. (d) *In situ* powder X-ray diffractograms collected upon exchange of ACN in **4** by DMF. (PXRD patterns were measred using radiation with λ =1.5406 Å.)

The other set of reflections, *i*.*e*., 004 and 040 remain at the same positions.

From this observation, it is reasonable to assume that in this period of time, the interlayer distance decreases upon exchange of ACN in a quasi-second-order phase transition to form an intermediate phase. No changes are visible until the new reflections appear at 2Θ values of 7.75°, 13.35° etc., in addition to those of **ip6**, and two sets of reflections coexist for the next 6 s (Region III, Figure 6a,b). At the 11th second of the experiment, the reflections of **ip6** disappear, and a new phase (compound **4**) is formed, which persists until the end of the experiment (for another 27 s, Region IV, Figure 6a,b).

In the time frame of the *in situ* PXRD experiment, the structural transition was reflected mainly in the “breathing” of the structure, indicating a strong decrease in the interlayer distance in the first phase, followed by the relaxation in the second phase of the transition.

Thus, the overall structural transition time is about 11 s, which is in agreement with the kinetics observed during *in situ* Raman spectroscopy measurements, showing that the exchange proceeds in less than 10 s. The solvent exchange in the pore is much faster, occurring almost instantaneously upon solvent arrival.

The *in situ* investigations of the reverse exchange from ACN back to DMF (from **4** to **5**) reveal shifts of one reflection, mainly corresponding to the further expansion of interlayer distance (Figure 6c,d; reflection marked in green). The zero time of the experiment was 257 s. A continuous shift of a 001 reflection at 2Θ = 8.7° can be observed over a time frame of 20 s, starting from 257 s and continuing until 277 s. The shorter period, compared to that obtained in *in situ* Raman spectroscopy measurements (30 s) can be explained by the lower time resolution in the Raman experiment, where the data can only be collected every 10 s.

The experiments demonstrate that the nature of the solvent influences the driving force and, consequently, the kinetics of terminal ligand exchange in DUT-134. The expansion of the interlayer distance appears as a continuous process (akin to a second-order phase transition).

The key factors governing structural transitions are the free energy differences between polymorphs as well as the kinetic barriers separating them. In the case of DUT-134, all experimentally observed solvated structures, independent of their stacking mode, transform into the same solvent-free structure with AA stacking (**1**, Figures S10 and S11), which represents the global minimum on the energy landscape of the framework. The stacking arrangements observed in the solvated compounds are therefore not an intrinsic characteristic of the empty DUT-134 framework but are stabilized by specific host–guest interactions. Structures with different stacking modes and interlayer distances correspond to local minima on the conformational energy landscape, separated by energy barriers associated with layer sliding or expansion. Transitions between these structures require crossing such barriers, which can be facilitated by stabilization through guest molecules. The adoption of a particular local minimum is driven by a reordering of the relative energies of these states, induced by molecules coordinating to the axial positions of the PW units and adsorbed within the pores.

Apparently, DUT-134_DMF (**2**, AB stacking) is more stable than DUT-134_ACN (AB conformation), and adding ACN to DUT-134_DMF (**2**) initiates the restacking to a more energetically favorable DUT-134_ACN (**4**, AA stacking). The rearrangement in the next exchange step to DUT-134_DMF (**5**, AA stacking) requires less energy than the transformation to **2**, hence we assume compound **5** to represent a trapped metastable state (local energetic minimum of DUT-134_DMF).

Also, the activation energy for the incorporation of ACN in AB stacking of **2** is lower than that of the incorporation of DMF into AA stacking of **4**. According to the Raman data discussed above, the exchange of DMF with ACN in the structure with AA stacking of the layers (**5** to **4** transition) is also significantly slower than the incorporation of DMF into AB stacking (**2** to **ip6** transition), confirming that the layer restacking as a structural transition accelerates the solvent exchange.

### Substrate Size Effect Analysis via In Situ Raman Spectroscopy and Longer Aliphatic Chain Nitriles

Raman spectroscopy was further utilized to investigate the influence of the chain length of aliphatic nitriles on the exchange kinetics in DUT-134. DMF in **2** was therefore exchanged by pentanenitrile or heptanenitrile, and the resulting shift in the Cu–O stretching vibration band was recorded over time. The band shifts from 406.7 cm^–1^ to 410.5 cm^–1^ for pentanenitrile and to 409 cm^–1^ for heptanenitrile (Figure 7).

**Figure 7:**
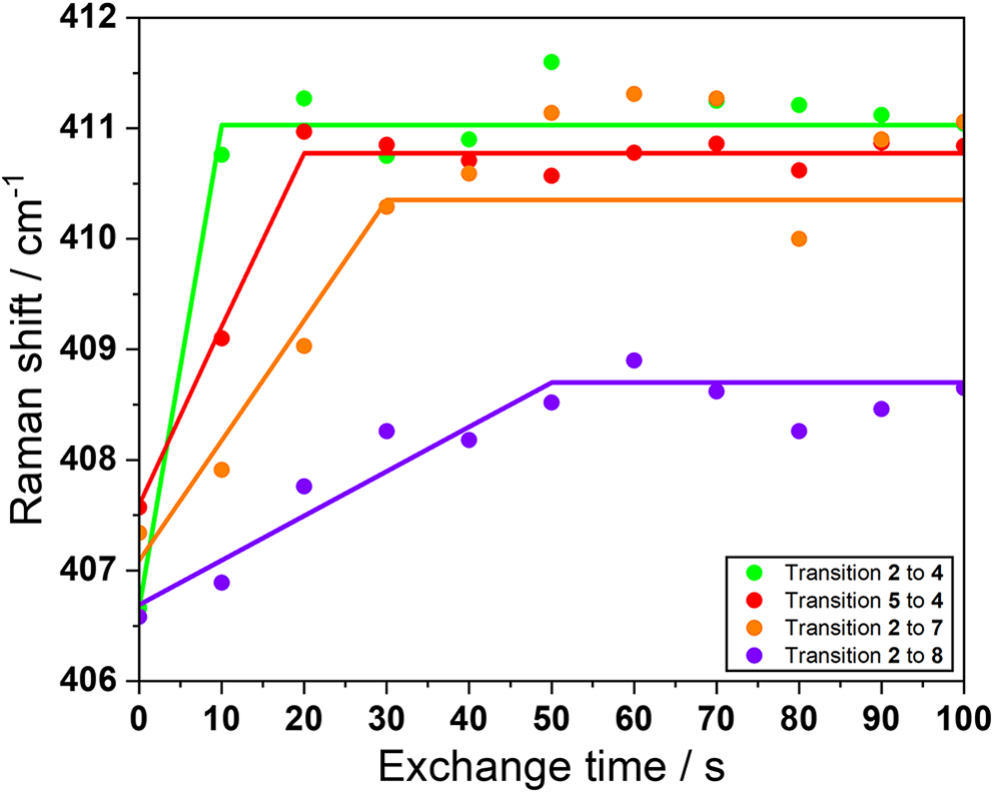
Solvent exchange kinetics of DMF-containing DUT-134 (**2**) with nitriles. Wavenumber of the Cu–O stretching Raman band during solvent exchange of DMF to ACN upon structural restacking from **2** to **4** (green) and without from **5** to 4 (red), exchange of DMF to pentanenitrile (**2** to **7**, orange), and to heptanenitrile (**2** to **8**, purple). The data are plotted starting from the respective zero time of the exchange experiment, which was determined by measuring changes in solvent Raman bands (see SI, Section 18).

The behavior of the Cu–O stretching vibration band is analogous to the behavior observed for ACN, but the band shifts became increasingly slower for nitriles with longer aliphatic chain lengths. The solvent exchange, accompanied by structural transition from **2** to **4** takes place within 10 s (Figure 7). The solvent exchange, which is not accompanied by a structural transition from **5** to **4** takes place within 20 s. For pentanenitrile approximately 30 s are needed, and around 50 s for heptanenitrile. This delay can be attributed to the reduced diffusion rate of these bulkier nitriles and to the higher activation energy required to adjust the interlayer distance. Cycling experiments were also conducted for ligand exchange with pentanenitrile and heptanenitrile. As it was previously shown for acetonitrile, it is also possible to cycle the ligand exchange process with longer alkyl chain nitriles as well.

For these exchanges, the kinetics remained consistent in multiple cycles: 30 s for pentanenitrile and 50 s for heptanenitrile (Figures S30 and S31).The results indicate that the exchange kinetics in DUT-134 are strongly governed by both the molecular size of the exchanging ligand and the mobility of the framework layers during phase transition. Larger ligands with longer alkyl chains, such as heptanenitrile and pentanenitrile, exhibit slower exchange rates due to a combination of steric hindrance within the interlayer space and reduced diffusion rates through the MOF channels. The consistent kinetics over multiple cycles indicate that the framework accommodates repeated ligand exchanges without degradation.

The obtained results are in accordance with self-diffusion coefficients of the analyte molecules. Self-diffusion of ACN has been widely studied.^70,71^ The diffusion of larger nitrile-containing analyte molecules is only scarcely discussed.^72^ However, looking at the self-diffusion of alkanes of different sizes in MOFs, a clear trend is observed: faster diffusion for molecules with shorter alkyl chains.^73,74^ These results are consistent with the herein-reported observations.

Based on the kinetics of the ligand exchange, it is assumed that pseudozero-order kinetics can play a role in this process. Since the amount of the exchanging solvent molecules is several orders of magnitude higher than that of the copper centers, the pseudozero-order approximation can be justified. Linear fits of the experimental data (see SI, Section 19) reveal that the exchange rate constant for pentanenitrile is approximately twice as low as that for acetonitrile, while that for heptanenitrile is about 4.5 times lower than for ACN. However, due to the rapid exchange process, the number of data points available for the fit is limited and must be handled with caution.

These findings lead to the conclusion that the temporal response of DUT-134 can be finely tuned by selecting ligands with specific sizes, polarities, or steric profiles, offering a strategy to control the interplay between ligand exchange, structural rearrangements, and framework symmetry. This mechanistic understanding provides a foundation for designing adaptive MOF-based catalysts and responsive materials, in which the kinetics of ligand exchange can be accelerated to increase reaction rates or manipulated to optimize selectivity and functional responsiveness.

## Conclusion

The temporal response at the second scale of the 2D metal–organic framework DUT-134­(Cu) during ligand exchange was analyzed via complementary *in situ* techniques, highlighting the dynamically coupled local symmetry changes and global stacking rearrangements induced by small donor molecules coordinating to open sites. The exchange of DMF by ACN triggers a symmetry increase from the *Pnma* to *P*4/*nmm* space group, and a significant structural transition, characterized by an increase in interlayer distance and a change in the layers’ stacking mode from AB to AA. This transition accelerates solvent exchange through a cooperative mechanism, reducing the exchange time to under ten seconds when accompanied by layer restacking, compared with about 20 s in the absence of structural rearrangement. The kinetics of ligand exchange were further explored for nitriles with longer alkyl chains, revealing slower exchange rates (30 s for pentanenitrile and 50 s for heptanenitrile) due to steric and diffusion constraints, as well as the absence of layer rearrangements. The use of *in situ* Raman spectroscopy and PXRD in a custom flow cell setup, complemented by isotopic labeling and computational simulations, enabled detailed assignment of Raman vibrational modes within the extended MOF structure and provided mechanistic insights into the exchange process. As the stacking mode of the layers dictates the electronic structure^75^ and charge transport properties,^76,77^ as well as influences the adsorption strength and adsorption selectivity^78^ in 2D MOFs, it consequently affects their catalytic behavior. Our findings underscore the potential of DUT-134 as a representative of widely explored paddle wheel-based 2D MOFs as adaptive materials for catalytic applications,^79^ indicating the interplay between structural flexibility and ligand dynamics in 2D MOFs, which can be generally harnessed to optimize performance, an important feature of precision chemistry in soft porous frameworks.

## Experimental Section

### Synthesis of **1** and **2**

Compound **2** and desolvated compound **1** were synthesized according to the procedure reported by Schwotzer et al. in 2021.^54^ For more details, see also Section 5 of SI.

### Synthesis of **3a** and **3b**

**3a** and **3b** were prepared following the synthesis protocol for **2**. The synthesis involved ^13^C–H_2_dttc (44 mg; 0.15 mmol; 1 equiv) or ^18^O–H_2_dttc (45 mg; 0.15 mmol; 1 equiv). The resulting dark green crystals were washed repeatedly with DMF.

### Synthesis of Compounds **4**, **5**, **7**, and **8**

A sample of **2** was subjected to an excess amount of the corresponding nitrile (acetonitrile in the case of **4**, pentanenitrile for **7**, or heptanenitrile for **8**) or DMF (in the case of **5**) for 3 days (Figure 2). The supernatant was replaced with a fresh one three times per day. All samples were characterized by PXRD and Raman spectroscopy.

### Single Crystal X-ray Analysis of **4** (DUT-134_ACN)

A single crystal of DUT-134, containing ACN in the pores, was placed into a borosilicate glass capillary (*d* = 0.3 mm) with a small amount of ACN. The data set was collected at the BL14.2 beamline of the BESSY II synchrotron, operated by Helmholtz–Zentrum Berlin für Materialien and Energie.^80^ Four images from different crystal orientations were collected in order to determine the crystal symmetry and scan angle range using the iMosflm program.^81^ The φ scan with an oscillation step of Δφ = 0.1° was used for the collection of 1800 frames, which were processed automatically using XDSAPP 2.0 software.^82^ The crystal structures were solved by direct methods and refined by full-matrix least-squares on *F* ^2^ using the SHELX-2018/3 program package.^83^ All non-hydrogen atoms were refined in an anisotropic approximation. Hydrogen atoms were refined in geometrically calculated positions using a riding model with *U*_iso_(H) = 1.2*U*_iso_(C).

### Crystallographic Data for Compound **4**

Cu_2_S_6_C_28_O_11_N_4_H_16_, *M* = 904 g·mol^–1^, tetragonal, *P*4/*nmm* (no. 129), *a* = 28.090(4) Å, *b* = 28.090(4) Å, *c* = 10.150(2) Å, *V* = 8009(3) Å^3^, *Z* = 4, *T* = 250 K, θ_max_ = 35.0°, reflections/parameter 346, *R*_int_ = 5.05, *R*_1_ = 7.02, *w*R**_2_ = 25.76, *S* = 1.041.

### Pawley Refinement of **5**

The PXRD pattern of **5** was analyzed using Materials Studio 5.0 software.^84^ The indexing of the pattern resulted in a tetragonal unit cell with cell parameters similar to those of the DUT-134_ACN structure (**4**). Therefore, the unit cell of **4** was used as an initial model for the Pawley fit, which shows a convergence between the calculated and theoretical profiles (see SI, Section 14).

### Crystallographic Data for Compound **5**

Tetragonal, *P*4/*nmm* (no. 129), *a* = 28.1131 Å, *b* = 28.1131 Å, *c* = 10.6663 Å, *V* = 8437.8 Å^3^, λ = 1.54059 Å, *T* = 296 K, 2θ_range_ = 5°–35°, profile function Thompson-Cox-Hastings, *U* = −0.00808, *V* = 0.01657, *W* = 0.00586, *X* = −0.43984, *Y* = 0.10889, *R*_p_ = 0.0372, and *R*_wp_ = 0.0486.

### Calculations of Raman Vibrational Modes for DUT-134

In the first stage (procedure I), the geometry of **1** ^54^ was optimized using density functional theory (DFT) as implemented in VASP (v5.4.4).^85–87^ Full optimization was considered here, including the volume, shape, lattice parameters, and ionic positions. The Perdew–Burke–Ernzerhof (PBE) functional was employed,^88^ including Grimme’s D3 dispersion correction with Becke–Johnson damping (D3BJ).^89^ A plane-wave energy cutoff of 600 eV was used, and calculations were performed at the Γ-point only. The convergence criteria were set to 10^–6^ eV for the SCF cycle (electronic energy) and 10^–2^ eV/Å for the geometry optimization steps (force). Spin polarization was included due to the open-shell nature of the Cu atoms. The total magnetic moment was set to enforce ferromagnetic ordering, which, although slightly higher in energy than the antiferromagnetic state (by approximately 0.04 eV per paddle wheel), preserves the crystal symmetry and is thus preferred for subsequent phonon calculations (see details below).

Phonon calculations were performed using the finite displacement method as implemented in Phonopy.^90,91^ A single supercell, sufficiently large to ensure accurate force constants,^92^ was used. The optimized structure belongs to the *P*2_1_/*m* space group, and symmetry analysis was employed to reduce the number of required single-point force calculations from 4,452 (no symmetry) to 484. Infrared intensities for phonon modes were computed using the Phonopy Spectroscopy module.^93^ A scaling factor of 1.02 ^94^ was applied to the calculated frequencies to minimize the deviation between experimental and simulated spectra. Born effective charges, necessary for IR intensity calculations, were obtained in VASP single-point calculations.

Cluster calculations based on a tetramer of four Cu-paddlewheels were performed in ORCA^95^ to analyze the magnetic ordering and spin states. The B3LYP hybrid functional^96,97^ was used in combination with the def2-TZVP basis set,^98^ with the def2/J auxiliary basis set^99^ and the RIJCOSX approximation to accelerate Coulomb and exchange integral evaluation. Tight SCF convergence criteria and the DEFGRID3 integration grid were applied. Spin analysis covered states with various spin polarizations, with antiferromagnetic ordering yielding the lowest energy. However, as noted above, the energy difference relative to the ferromagnetic state is minor (∼0.04 eV per paddle wheel), and the ferromagnetic configuration was used for simplicity in phonon simulations.

The procedure II for calculating the Raman spectra of **2** was adopted from the methodology described by Bas et al.^100^ and is briefly summarized below. All DFT calculations were performed using the CP2K software package at the Γ-point only with a supercell containing 512 atoms.^101^ The PBE exchange-correlation functional with D3 dispersion correction,^102^ Goedecker–Teter–Hutter pseudopotentials,^103–105^ and the DZVP-MOLOPT-SR basis set were used in all calculations.^106^

In the first step, the structure of **2** was tightly optimized without optimizing the cell parameters. Afterward, 6N geometries were generated by displacing each atom of the optimized structure by ±0.001 Å in the *x*, *y*, and *z* directions, respectively. For each of the 6N displaced structures, the forces were calculated to numerically construct the mass-weighted Hessian matrix via a central finite difference approach. The mass-weighted Hessian matrix was used to determine the normal-mode frequencies and normal mode displacements. To obtain Raman intensities, the polarizability tensor for each of the 6N displaced structures was calculated using density functional perturbation theory (DFPT) as implemented in the CP2K software.^107^ The Raman intensities were then determined from the first derivatives of the polarizabilities along the mass-weighted normal mode coordinates. The unpolarized intensities were generated at an incident laser wavelength of 523 nm. To simplify the comparison with the experiment, the final calculated Raman spectrum of **2** was obtained using Gaussian broadening. A data set with the input and output files of the calculations is available on Zenodo.^108^ The comparison of the obtained data is provided in Section 11 of the SI.

The same workflow, as described for **2** (procedure II) was also applied to compute the Raman spectra of **1**, using the FHI-aims software package.^109^ All FHI-aims calculations were performed using the PBE exchange-correlation functional^88^ with the Tkatchenko-Scheffler dispersion correction.^110^ Numerical atom-centered orbitals of *tier1* and *tier2* quality were used in combination with “intermediate” numerical settings.^109^ A 2 × 1 × 1 *k*-point grid with 320 atoms in the unit cell was used. For the smaller system, cell parameter optimization was performed in addition to the tight geometry optimization, and the 6N displaced structures were generated by a shift of ±0.0025 Å. In all other respects, the procedure is the same as for system **2**.

## Supplementary Material



## Data Availability

Experimental data discussed in this publication can be accessed through a Zenodo repository.^66^ Another Zenodo repository contains data on the calculations of vibrational spectra.^108^

## Notes

Materials and methods, synthetic procedures for linker and MOF isotope labeling, crystal structure determination details, vibrational spectroscopy of starting compounds, Raman band assignment, SEM, *in situ* PXRD background analysis, and Raman spectroscopy of pentanenitrile and heptanenitrile (PDF)

## Abbreviations

ACN: acetonitrile
BFDH: Bravais–Friedel–Donnay–Harker
COF: covalent-organic framework
DEPT: Density Functional Perturbation Theory
DFT: Density Functional Theory
DMF: *N,N*-dimethylformamide
DMSO: dimethyl sulfoxide ip intermediate phase
dttc: dithieno­[3,2-b:2′,3′-*d*]­thiophene-2,6-dicarboxylate
DUT: Dresden University of Technology
MOF: metal–organic framework
PBE: Perdew–Burke–Ernzerhof
PW: paddle wheel
PXRD: powder X-ray diffraction
2D: two-dimensional
3D: three-dimensional
