# Supplementary material for: In Situ Insights into Enhanced Cooperative Ligand Exchange Kinetics via Solvent-Induced Restacking in a 2D Metal–Organic Framework
Source: J Am Chem Soc. 2026 Mar 30;148(13):13940–53. doi: 10.1021/jacs.5c22455 (PMC13067260; doi:10.1021/jacs.5c22455)
Supplement: Supplementary file 1 [file ja5c22455_si_001.pdf]

Electronic Supporting Information

for

In situ Insights into Enhanced Cooperative Ligand Exchange Kinetics via Solvent-Induced Restacking in a 2D Metal-Organic Framework

Richard Engemann[a], Irena Senkovska*[a], Friedrich Schwotzer[a], Josefine Winkler[b], Volodymyr Bon[a], Susanne Machill[b], Philipp Wollmann[c], Fanny Reichmayr[c], Jonas Weiß[a], Johannes Scheffler[d], Ekin Esme Bas[d], Filip Formalik[e,f], Randall Q. Snurr[e], Dorothea Golze[d], Inez M. Weidinger[c], Eike Brunner[b], Stefan Kaskel*[a]

[a] Chair of Inorganic Chemistry I, Technische Universität Dresden, 01069 Dresden, Germany.

[b] Chair of Bioanalytical Chemistry, Technische Universität Dresden, 01069 Dresden, Germany.

[c] Chair of Electrochemistry, Technische Universität Dresden, 01069 Dresden, Germany.

[d] Chair of Theoretical Chemistry, Technische Universität Dresden, 01069 Dresden, Germany.

[e] Department of Chemical & Biological Engineering, Northwestern University, 2145 Sheridan Road, Evanston, Illinois 60208, United States.

[f] Department of Micro, Nano and Bioprocess Technology, Wroclaw University of Science and Technology, 50-307 Wroclaw, Poland.

Table of contents

1. Materials and methods 3

 1.1 Materials used 3

 1.2 Ex situ Raman spectroscopy 3

 1.3 In situ Raman spectroscopy in a flow cell apparatus 3

 1.4 ^{1}H- and ^{13}C-NMR spectroscopy 4

 1.5 Powder X-ray diffraction (PXRD) 4

 1.6 In situ powder X-ray diffraction in the flow cell apparatus 4

 1.7 High resolution time-of-flight mass spectrometry (HR-TOF-MS) 5

 1.8 Elemental analysis (EA) 5

2. Synthesis of ^{13}C-isotope labelled H_2dttc 6

3. Synthesis of ^{18}O-isotope labelled H_2dttc 6

4. ^{1}H- and ^{13}C-NMR spectroscopy of ^{13}C-H_2dttc and ^{18}O-H_2dttc 7

5. Synthesis of compounds 2 and 1 9

6. Synthesis of ^{13}C- and ^{18}O-isotope labelled 3a and 3b 10

7. Powder X-ray diffraction patterns of 1, 2, 4, 5, 7 and 8 11

8. Single crystal X-ray crystallography of 4 14

9. Density calculations for solvent-filled compounds 2, 4 and 5 15

10. IR and Raman spectra of H_2dttc and 1 16

11. Theoretical IR and Raman spectra of solvent-free DUT-134 21

12. Raman spectra of 2, 4, 5, 7 and 8 23

13. Scanning Electron Microscopy (SEM) 24

14. Pawley refinement of compounds 5, 7 and 8 24

15. Analysis of *in situ* PXRD amorphous background for dead time determination 27

16. Script for analysis of PXRD amorphous background 28

17. Cu-O Raman band shift upon solvent exchange 29

18. Ligand exchange kinetics for acetonitrile, heptanenitrile, and pentanenitrile 32

1. Materials and methods

1.1 Materials used

All the reagents and solvents for synthesis were purchased from commercial sources and used as supplied without further purification.

Table S1. List of chemicals used for the synthesis of **1** - **8**.

Chemical name	CAS number	Purity	Manufacturer
Acetonitrile	75-05-8	99.95 %	Chemsolute
$^{13}CO_2$ gas	1111-72-4	99 % ^{13}C	Eurisotop
$C^{18}O_2$ gas	124-38-9	95 % ^{18}O	Eurisotop
Copper(II)nitrate trihydrate	10031-43-3	98 %	Sigma Aldrich
2,6-Dibromodithieno[3,2-b:2',3'-d]thiophene	67061-69-2	>98 %	TCI Europe
Dimethylsulfoxide	67-68-5	99.9 %	Fisher Scientific
Dithieno[3,2-b:2',3'-d]thiophene-2,6-dicarboxylic acid	502764-53-6	97 %	BLD Pharm
Ethanol	64-17-5	99.8 %	Sigma Aldrich
Heptanenitrile	629-08-3	98 %	Fisher Scientific
Hydrochloric acid	7647-01-0	37 %	Sigma Aldrich
n-Butyllithium	109-72-8	2.5 M in hexanes	Sigma Aldrich
N,*N*-Dimethylformamide (DMF)	109-72-8	99.5 %	Fisher Scientific
Tetrahydrofuran (THF)	109-99-9	≥99 %	Sigma Aldrich
Pentanenitrile	110-59-8	99 %	Fisher Scientific

1.2 Ex situ Raman spectroscopy

Raman measurements were performed on a Confocal Raman Microscope (CRS+) MonoVista from Spectroscopy & Imaging GmbH equipped with a liquid nitrogen cooled CCD detector (PyLoN 100BR_excelon, Princeton Instruments) at the laser excitation wavelengths of 514 nm (Cobolt Fandango) and 785 nm (IPS Single-Mode D-Type). The lasers were focused on the sample surface using a Nikon 20x objective (0.35 NA, 20 mm WD), and the backscattered light at 180° was detected. This experimental setup was used to record the data discussed in Figures 3 and 4.

1.3 In situ Raman spectroscopy in a flow cell apparatus

Approximately 5 milligrams of DUT-134 crystals were immersed in an organic solvent (in this case, *N,N*-dimethylformamide (DMF) or nitrile) and loaded into a constricted glass capillary (0.80 mm in diameter, narrowed to 0.15 mm). The capillary was then filled with glass fibers adjacent to the sample to immobilize the crystals and subsequently equipped with a 0.5 mm-thick silicone hose at both sites. The exchanging solvent was supplied through the flow cell at a flow rate of 0.2 $mL \cdot min^{-1}$ using a KDS Legato 110 single syringe infusion/withdrawal pump. Concurrently, Raman spectra were recorded at an interval of 10 seconds for a total duration of 2 minutes using a DXR

Smart Raman spectrometer from Thermo Fisher Scientific. The laser wavelength utilized was 532 nm, and the beam diameter was set at 2.1 µm. The spectra were captured by a CCD detector, and a wide range spanning from 3500 cm^{-1} to 100 cm^{-1} with a resolution of 5 cm^{-1} was employed.

The ligand exchange process at the copper center was analyzed by monitoring the Cu-O vibration at approximately 407 cm^{-1} over time. Based on the bands with the highest intensity in the solvent spectra, the relative intensities of the N-CH_3 stretch of DMF at 860 cm^{-1} and the N≡C stretch of ACN at 2251 cm^{-1} were used to detect the solvents inside the flow cell.

This experimental setup was used to record the data discussed in Figures 5 and 7.

1.4 ^{1}H- and ^{13}C-NMR spectroscopy

Nuclear magnetic resonance (NMR) spectra were measured on a BRUKER DRX 500 P spectrometer (500.13/600.16 MHz and 125.77/150.91 MHz for ^{1}H and ^{13}C, respectively) and/or on a BRUKER AC 300 P (300 MHz, 282 MHz, and 75.5 MHz for ^{1}H and ^{13}C, respectively). All ^{1}H- and ^{13}C-NMR spectra are reported in parts per million (ppm) downfield of TMS and were measured relative to the residual signals of the solvents at 7.26 ppm ($CDCl_3$) and 2.50 ppm (DMSO). Data for ^{1}H-NMR spectra are described as follows: chemical shift [δ (ppm)], multiplicity (s, singlet; d, doublet; br, broad signal), coupling constant J (Hz). Data for ^{13}C -spectra are described in terms of chemical shift [δ (ppm)], and functionality was derived from DEPT (Distortionless Enhancement by Polarization Transfer) spectra.

1.5 Powder X-ray diffraction (PXRD)

PXRD measurements at room temperature were performed on an STOE STADI P diffractometer using Cu $K\alpha_1$ radiation (λ = 1.5405 Å) and a 2D detector (Mythen, Dectris) in transmission geometry. Step scans with 120 s/step and a detector step size of 3° were used for data collection. The samples were placed between non-diffracting MPET foils (rescue blanket). Activated samples were prepared in inert atmosphere (in an argon-filled glove box). Theoretical PXRD patterns were calculated from single-crystal structure data using the Mercury 4.0 software package[1].

1.6 In situ powder X-ray diffraction in the flow cell apparatus

Time-resolved *in situ* PXRD experiments were conducted at P23 diffraction and imaging beamline of PETRA III synchrotron (DESY). A monochromatic parallel X-ray with E = 20.0 keV (λ = 0.619921 Å) and a size of 400x100 µm was used in all experiments. Reflection intensities were measured using PILATUS 1M (DECTRIS) detector, installed 45 cm back from the sample. In the typical experiment, PXRD patterns were collected at 10 Hz rate. In parallel, the optical images were collected using a motorised OptoSigma microscope (ZLM-181041) equipped with Allied Vision Mako G-419C camera.

The experimental setup consists of the syringe pump (KDS Legato 110 single syringe infusion/withdrawal pump) connected to the customized home-built *in situ* glass capillary adsorption cell. A glass capillary, with a diameter of about 700 µm was connected from the top through a tube to the syringe pump. The analyte material is placed in the middle of the capillary

and fixed with quartz wool. Acetonitrile with a flow rate of 0.2 mL·min^{-1} was pumped through the capillary at ambient temperature (298 K). The contact time of the solvent with the powder positioned in the synchrotron beam was determined from the changes in the background scattering of the powder X-ray diffraction patterns.

In order to analyze the exchange kinetics, it is essential to estimate the "zero time", defined as the moment when the analyte comes into contact with the sample. To this end, the background scattering data obtained from diffraction experiments were fitted with a function, specifically a background fit, using the "peakutils.baseline" algorithm in a Python script[2]. This baseline estimation algorithm involves the iterative fitting of a polynomial to the data to detect its baseline. In each iteration, the fitting weights on the regions with peaks are reduced to identify the baseline only. The second derivative changes between subsequent diffractograms were plotted against the measuring time to clarify the exact moment the solvent hits the crystals (aka zero time). The result of this action is a sharp increase in the second derivative for the next baseline function.

1.7 High resolution time-of-flight mass spectrometry (HR-TOF-MS)

High resolution time-of-flight mass spectrometry (HR-TOF-MS) was carried out on a maXis TOF of Bruker. The samples were dissolved in dimethyl sulfoxide or ethyl acetate and diluted with a 1% ammonium formate solution in methanol. The solution was ionized by electro-spray ionization.

1.8 Elemental analysis (EA)

Elemental analysis was performed on a HEKAtech EA 3000 Euro Vector CHNSO Elemental Analyzer in CHNS mode. The composition was determined as the average of three individual measurements on three individually prepared samples. Desolvated crystals of DUT-134 were analyzed by placing 3-5 mg of sample in tin sample holders under an inert atmosphere in a glove box and sealed to avoid contamination by humidity.

2. Synthesis of ^{13}C-isotope labelled H_2dttc

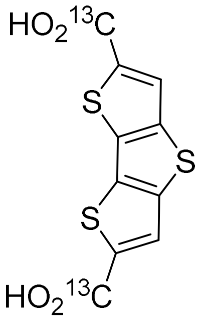

2,6-Dibromodithieno[3,2-b:2',3'-d]thiophene (310 mg, 0.88 mmol; 1.00 eq.) was placed into a dry 50 mL Schlenk flask under argon atmosphere. Subsequent synthetic steps were performed under inert conditions using a Schlenk line. The solid was dissolved in 15 mL of degassed, anhydrous THF. The light yellow, almost colorless solution was then cooled to -78 °C and stirred at this temperature for 30 min. Subsequently, *n*-BuLi (0.75 mL, 1.88 mmol; 2.5 M; 2.15 eq.) was added slowly over 10 min. The resultant yellow-beige colored suspension was then warmed up to 0 °C and stirred for 10 min. The labelled $^{13}CO_2$ gas was then introduced to the suspension, exchanging the Ar atmosphere. This resulted in a local green coloration, which disappeared again after a few minutes. The yellow-beige colored suspension was stirred under slight CO_2 overpressure for 12 h and was gradually warmed to room temperature (RT). To work up the reaction, 10 mL of H_2O was added to deactivate any unreacted *n*-BuLi. Subsequently, the rotary evaporator was employed to completely remove THF, and 4 mL HCl (6 M) was added to the solution, resulting in the precipitation of a greenish solid. The obtained crystalline product was washed multiple times with water and a small amount of EtOH, followed by drying at 60 °C.

Yield: 229 mg (0.78 mol; 89 %). The purity was verified by ^{1}H-NMR, ^{13}C-NMR, and ESI-MS.

^{1}H-NMR (500 MHz, DMSO-d_6) δ (ppm): 8.24 (d, *J* = 2,6 Hz, 2 H), 13.50 (br, s, 2 H). ^{13}C-NMR (151 MHz, DMSO-d_6) δ (ppm): 127.63 (s, 2 CH), 136.03 (s, 2 C), 137.13 (s, 2 C), 143.62 (s, 2 C), 163.13 (s, 2 C). ESI-MS (m/z): Calculated for $C_8{}^{13}C_2H_4O_4S_3$: 285,93; found: 239,9326. $[M\text{-}H\text{-}^{13}CO_2]^-$, 194,9394 $[M\text{-}H\text{-}^{13}C_2O_4]^-$.

3. Synthesis of ^{18}O-isotope labelled H_2dttc

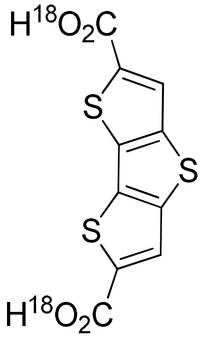

The synthesis of the ^{18}O-labelled H_2dttc linker was conducted in accordance with the procedure described in section 2 of the ESI for ^{13}C-H_2dttc employing ^{18}O-enriched carbon dioxide as the reactant.

Yield: 211 mg (0.74 mol; 84 %). The purity of the product was confirmed by ^{1}H-NMR, ^{13}C-NMR, and ESI-MS.

^{1}H-NMR (500 MHz, DMSO-d_6) δ (ppm): 8.24 (s, 2 H), 13.50 (br, s, 2 H). ^{13}C-NMR (151 MHz, DMSO-d_6) δ (ppm): 127.61 (s, 2 CH), 134.14 (s, 2 C), 136.56 (s, 2 C), 143.61 (s, 2 C), 162.98 (s, 2 C). ESI-MS (m/z): Calculated for $C_{10}H_4{}^{18}O_4S_3$: 291,94; found: 242,9379 $[M\text{-}H\text{-}C^{18}O_2]^-$, 191,9395 $[M\text{-}H\text{-}C_2{}^{18}O_4]^-$.

4. ^{1}H- and ^{13}C-NMR spectroscopy of ^{13}C-H_2dttc and ^{18}O-H_2dttc

All peaks in the ^{1}H- and ^{13}C-NMR spectra of the ^{13}C- and ^{18}O-isotope labelled H_2dttc could be assigned to the individual atoms, confirming the successful synthesis of the dicarboxylic acids.

The ^{13}C-NMR spectrum of ^{13}C-H_2dttc (Fig. S2) is particularly noteworthy in this regard, as it shows a pronounced intensity for the carboxylate carbon peak at 163 ppm. The doublet peak at 8.24 ppm also indicates successful incorporation of the ^{13}C-labelled moiety, due to long-range ^{1}H-^{13}C-coupling. These findings further confirm the successful carboxylation reaction facilitated by the isotope-labelled $^{13}CO_2$ used. Furthermore, ESI-MS analysis of ^{13}C-H_2dttc shows a fragment at 239.93 m/z, which corresponds to the monocarboxylate radical. A similar fragment was observed for ^{18}O-H_2dttc at 242.94 m/z, thereby substantiating the carboxylation of the dibrominated dtt core.

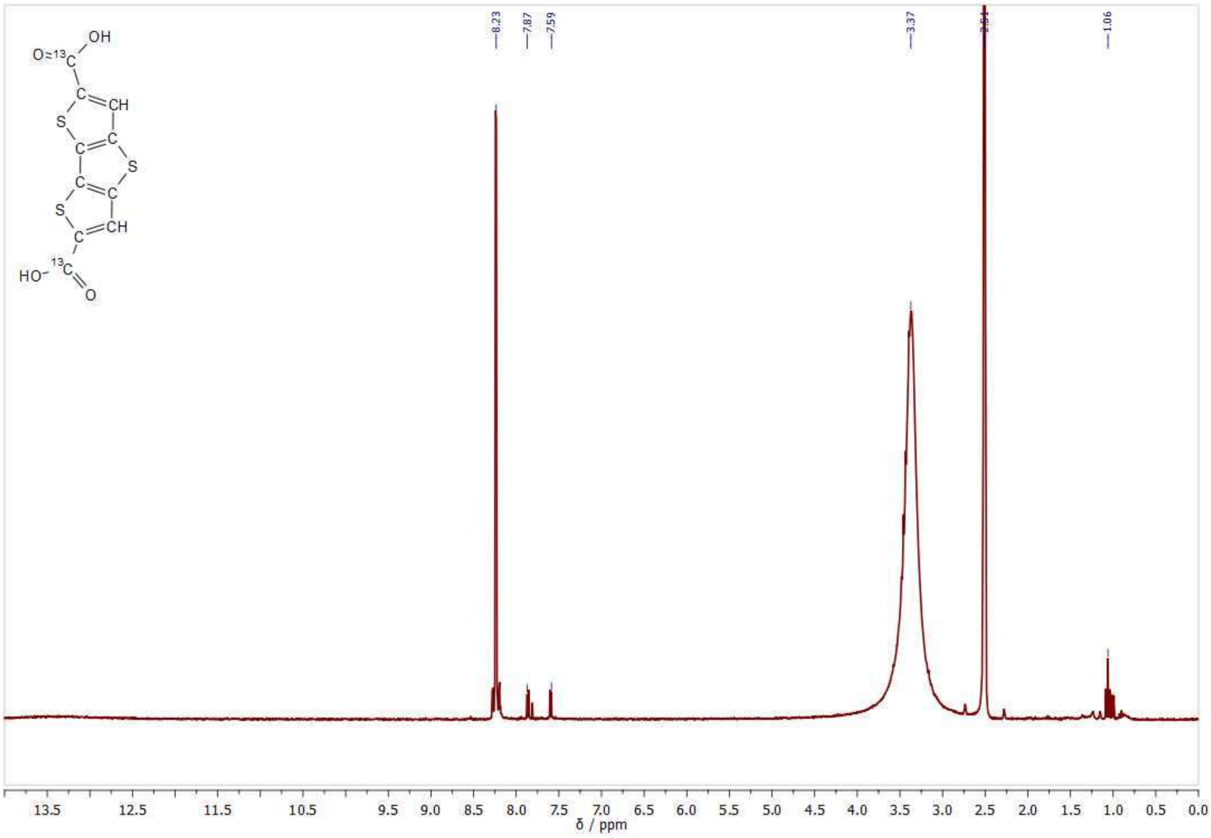

Figure S1. ^{1}H-NMR spectrum of ^{13}C-H_2dttc in DMSO-d_6.

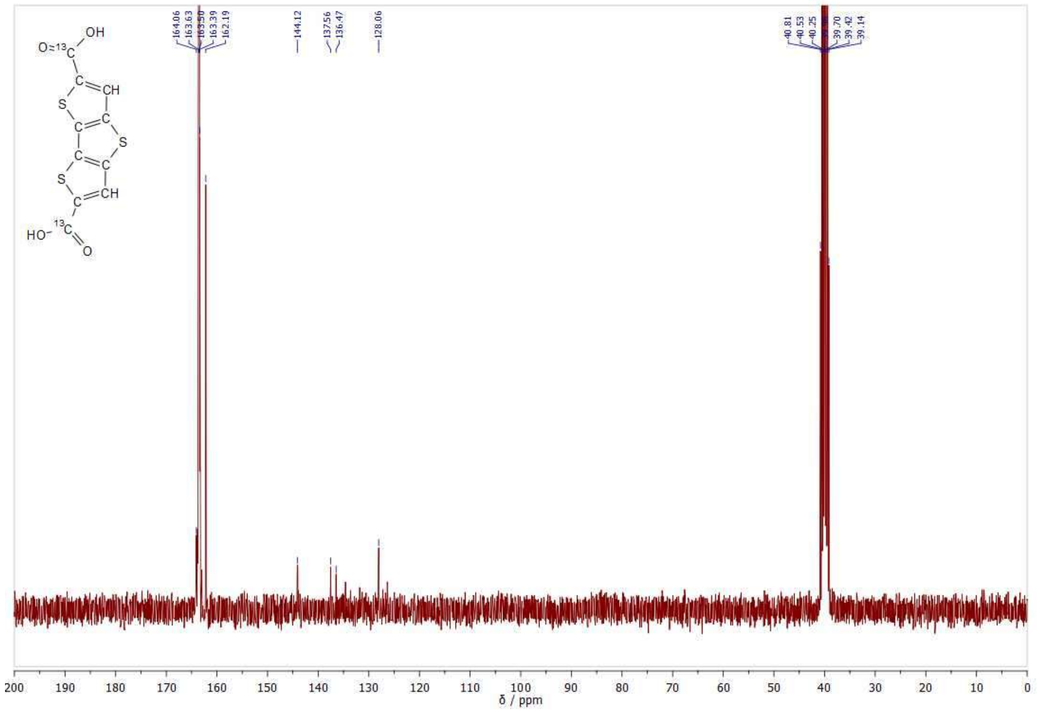

Figure S2. ^{13}C-NMR spectrum of ^{13}C-H_2dttc in DMSO-d_6.

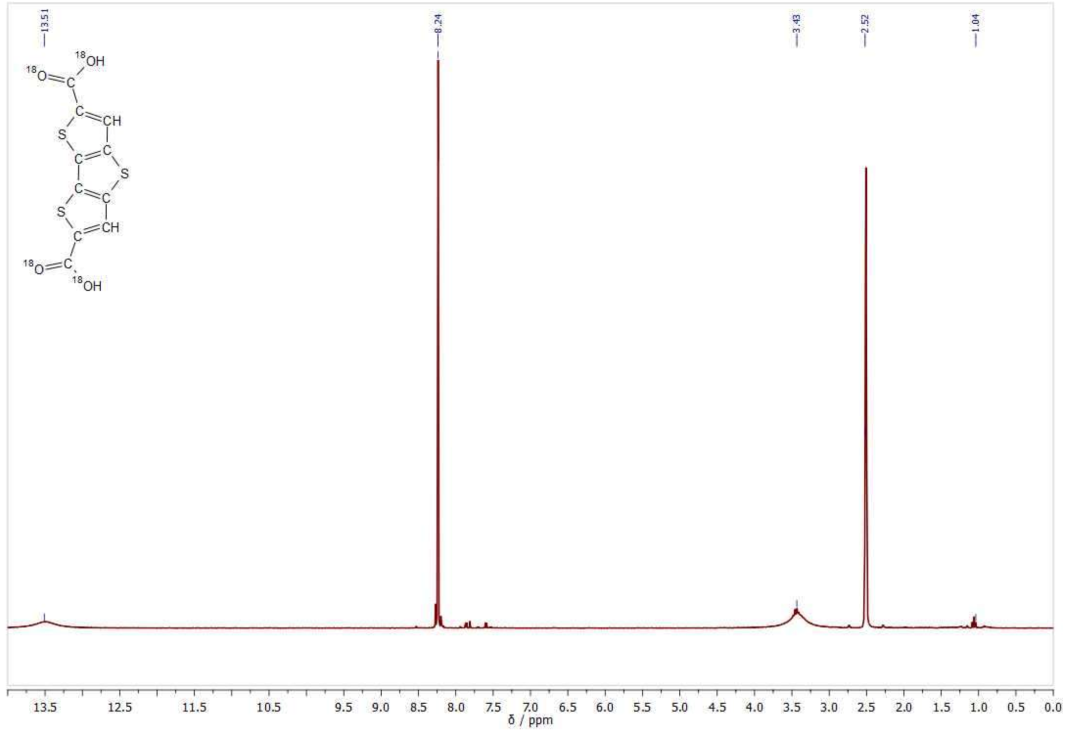

Figure S3. ^{1}H -NMR spectrum of ^{18}O-H_2dttc in DMSO-d_6.

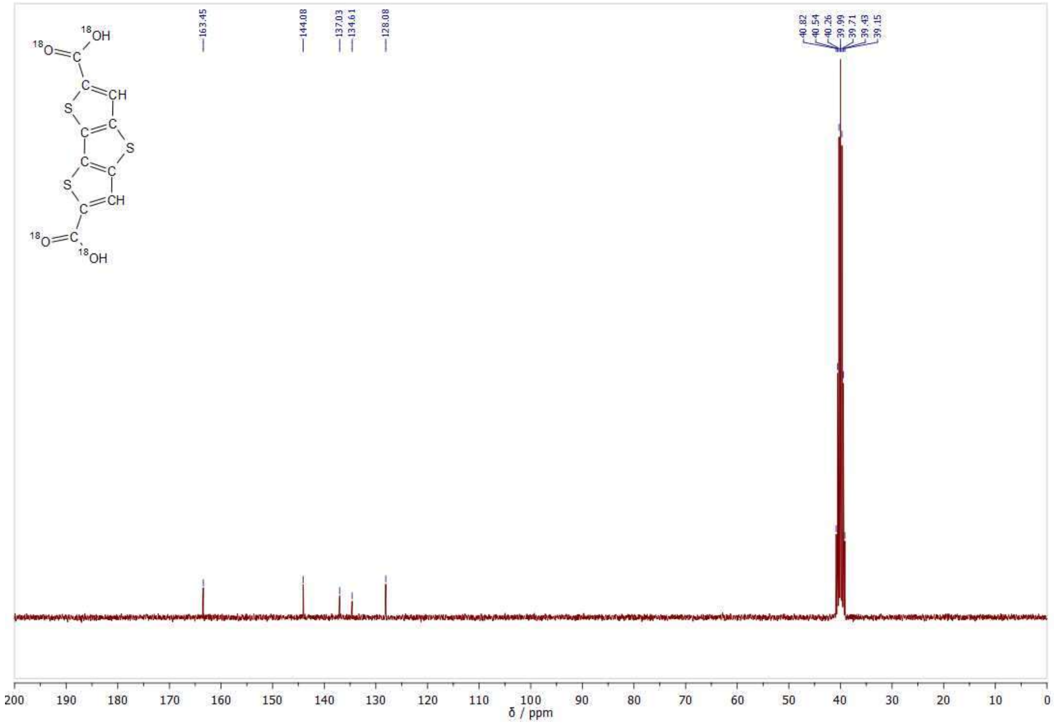

Figure S4. ^{13}C-NMR spectrum of ^{18}O-H_2dttc in DMSO-d_6.

5. Synthesis of compounds 2 and 1

Compound **2** was synthesized according to the procedure reported by Schwotzer *et al.* in 2021[3].

H_2dttc (43.5 mg; 0.15 mmol; 1.0 eq.) was dissolved in 6.7 mL DMSO using an ultrasonic bath and placed in a 25 mL Teflon insert of an autoclave. Subsequently, 6.7 mL of a DMSO/EtOH mixture (1:1) was overlaid before a solution of $Cu(NO_3)_2 \cdot 3H_2O$ (67.0 mg; 0.28 mmol; 1.8 eq.) in 6.7 mL EtOH (abs.) was added. The autoclave was first annealed at 60 °C for 72 h, after which it was heated at 90 °C for six days. The resultant dark green crystals approx. 200 µm in size (Fig. S13) were decanted from the reaction solution and thoroughly washed multiple times with DMF.

The crystals of **2** were desolvated using supercritical CO_2, yielding **1,** following the protocol reported of Schwotzer *et al.*[3] The desolvated samples were stored in a glovebox under Ar atmosphere.

Elemental analysis for **1**: Calculated (for $Cu_2(dttc)_2$): C: 34.73%; H: 0.58%; N: 0%; S: 27.81%; found: C: 34.61%; H: 0.57%; N: 0%; S: 27.71%.

6. Synthesis of ^{13}C- and ^{18}O-isotope labelled 3a and 3b

The isotope-labelled variants of DUT-134_DMF (**3a**/**3b**) were prepared in accordance with the synthesis description for **2**, as described in Section 5 ESI. The synthesis involved the use of ^{13}C-H_2dttc (44 mg; 0.15 mmol; 1 eq.) or ^{18}O-H_2dttc (45 mg; 0.15 mmol; 1 eq.). The resulting dark green crystals were washed repeatedly with DMF.

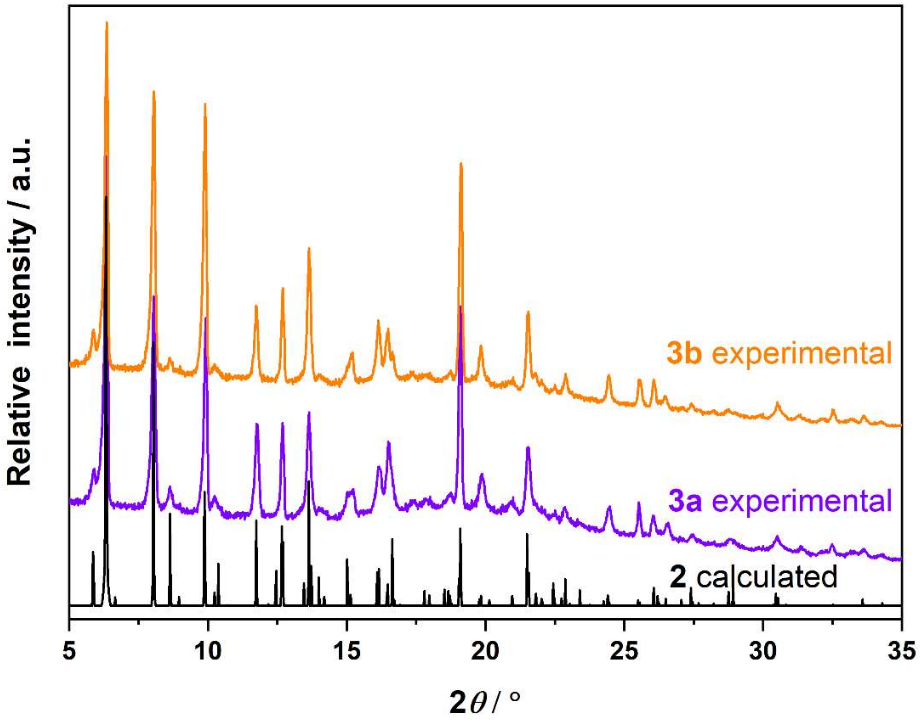

Figure S5. PXRD patterns of **3a** (purple), **3b** (orange) and calculated from the crystal structure of DUT-134_DMF[3] (CCDC 2069415, **2**, black).

7. Powder X-ray diffraction patterns of 1, 2, 4, 5, 7 and 8

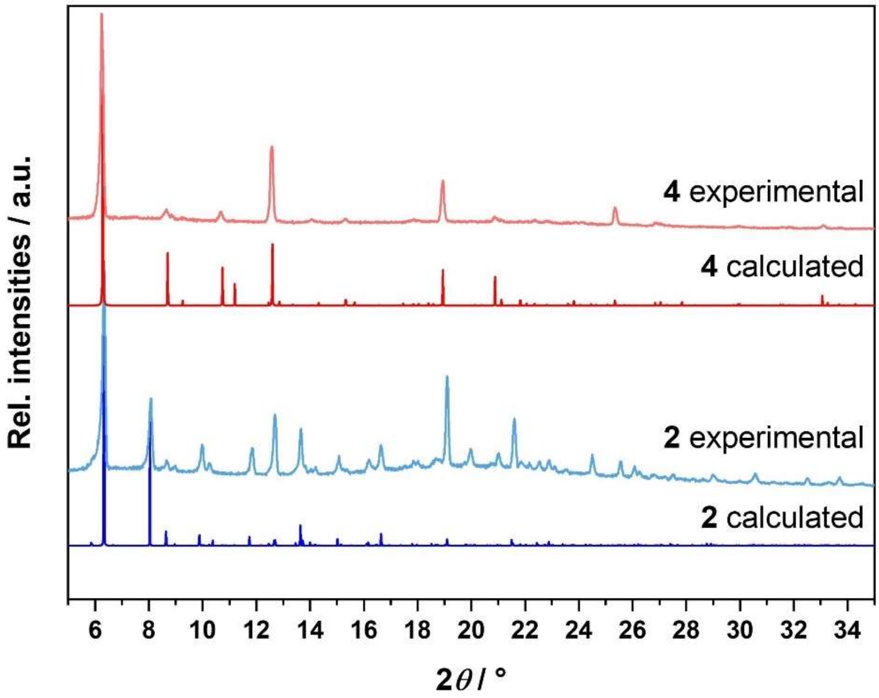

Figure S6. PXRD patterns of **2** (calculated in dark blue, experimental in light blue) and **4** (calculated in dark red, experimental in light red).

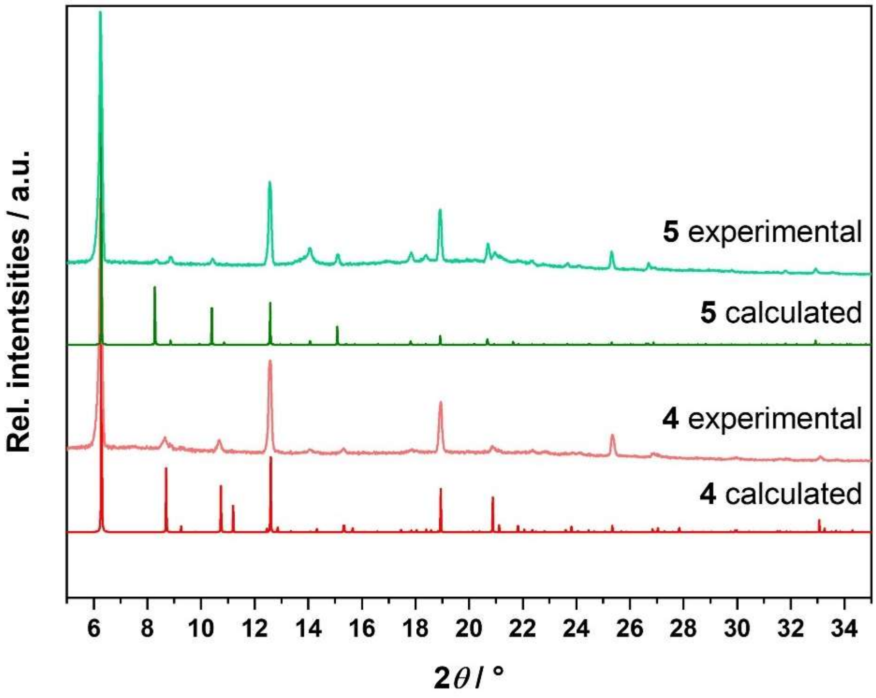

Figure S7. PXRD patterns of **4** (calculated in dark red, experimental in light red) and **5** (calculated in dark green, experimental in light green).

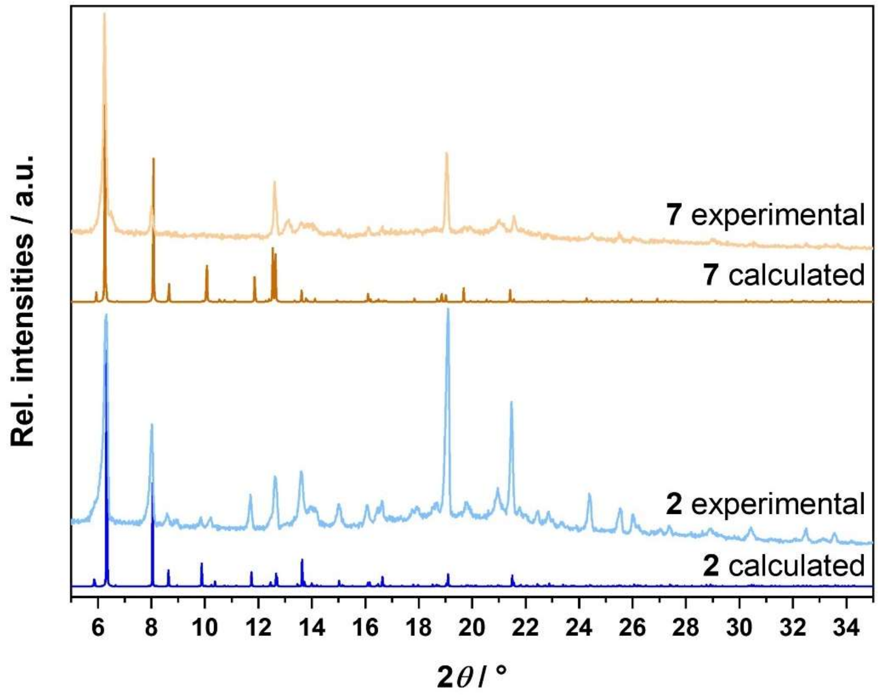

Figure S8. PXRD patterns of **2** (calculated in dark blue, experimental in light blue) and **7** (calculated in dark orange, experimental in light orange).

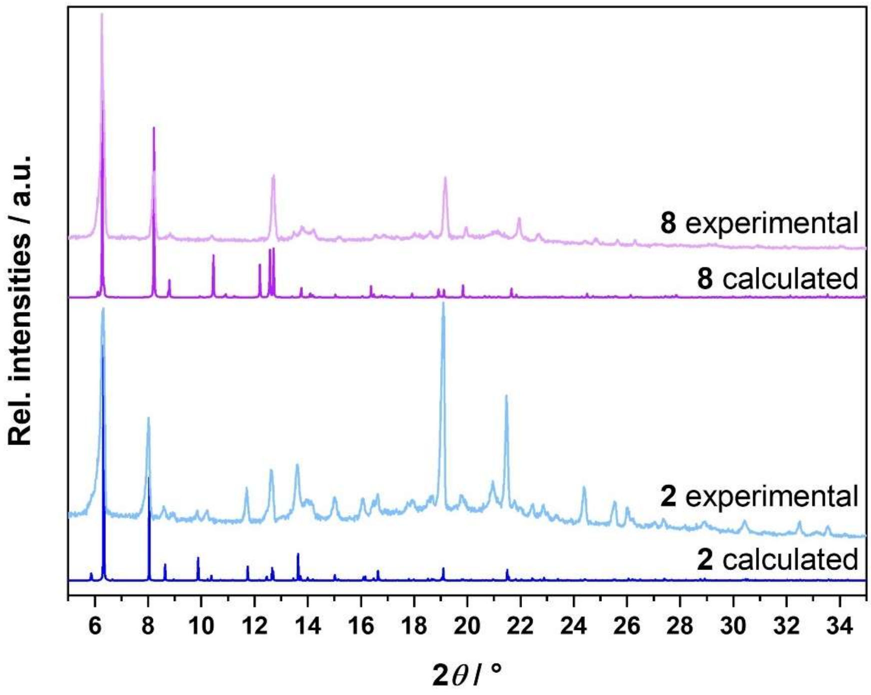

Figure S9. PXRD patterns of **2** (calculated in dark blue, experimental in light blue) and **8** (calculated in dark purple, experimental in light purple).

Activation of **2** to **1** was performed through an exchange of coordinated DMF by acetone through immersion of the crystals in pure acetone. In an autoclave, acetone was replaced by liquid CO_2, which was removed by means of supercritical drying. The resulting product is named **1**. Immersing crystals of **1** in DMF does not show a phase transition back to **2**, even after three days.

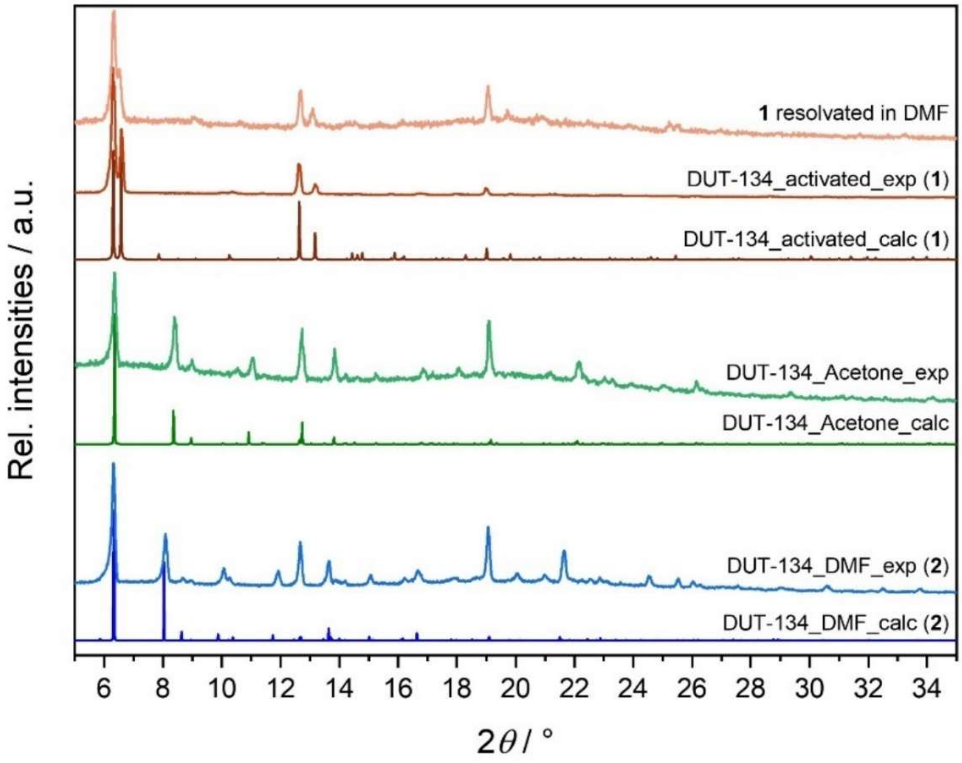

Figure S10. PXRD patterns of **2** (calculated in dark blue, experimental in light blue), DUT-134 coordinated by acetone (calculated in dark green, experimental in light green), **1** (calculated in dark brown, experimental in light brown) and **1** after resolvation of the framework with DMF (lighter brown), which does not lead to a phase transition back to **2**.

Activation of **4** to **1** was performed in an autoclave, where the coordinating acetonitrile was replaced by liquid CO_2, which was again removed via supercritical drying. In this case, the resulting crystal structure is also **1**. Immersing the crystals of **1** in CAN does show a phase transition back to **2**, concluding that ACN is able to reverse the phase transisiton to **1** during activation, while DMF is not able to provoke this phase transition.

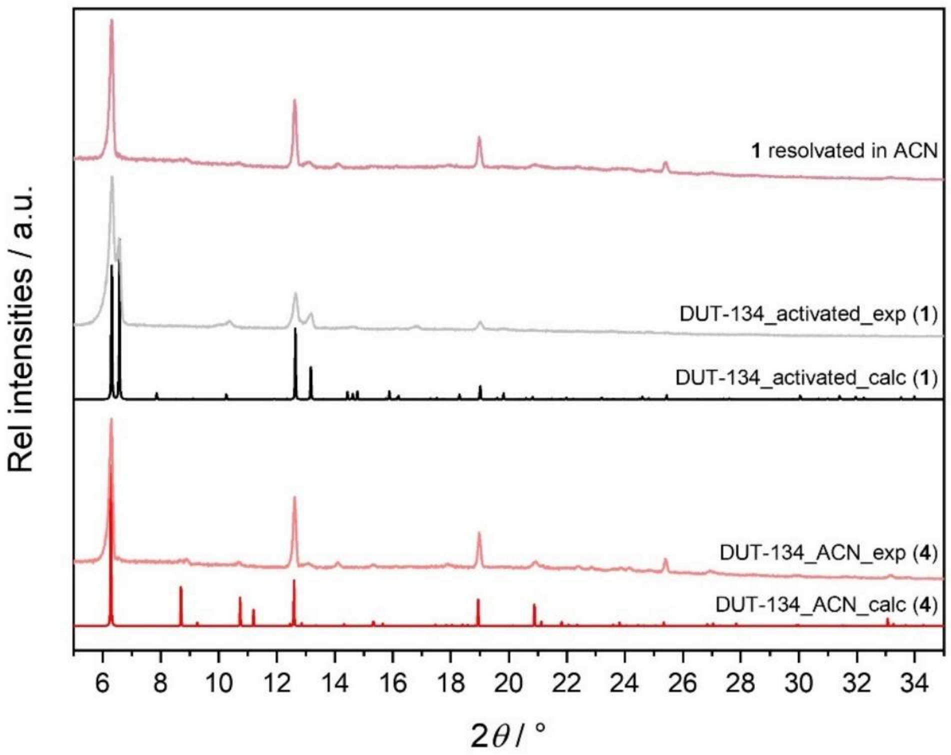

Figure S11. PXRD patterns of **4** (calculated in dark red, experimental in light red), **1** (calculated in black, experimental in grey) and **1** after resolvation of the framework with ACN (lighter red), which leads to a phase transition back to **2**.

8. Single crystal X-ray crystallography of 4

Table S1. Crystallographic data for DUT-134_ACN (**4**, CCDC 2500512).

	DUT-134_ACN (**4**)
Empirical formula	$Cu_2S_6C_{28}O_{11}N_4H_{16}$
Formula weight [g·mol^{-1}]	900.690
Crystal system, space group	Tetragonal, *P4/nmm* (no. 129)
Unit cell dimensions [Å]	*a* = 28.090(4); *b* = 28.090(4); *c* = 10.150(2)
Volume [Å^3]	8009(3)
Z	4
Absorption coefficient	1.079
F(000)	1810
Θ range [deg.]	1.19 - 35.01
Limiting indices	-30 ≤ *h* ≤ 30; -37 ≤ *k* ≤ 37; -11 ≤ *l* ≤ 3
Reflections collected / unique / R(int)	62471 / 5725 / 0.051
Data / parameters	5725 / 143
GooF on F^2	1.041
Final *R* indices	R_1 = 0.0702, wR_2 = 0.2576
Largest diff. peak / hole [e·Å^{-3}]	0.640 / -0.285

9. Density calculations for solvent-filled compounds 2, 4 and 5

The density of the solvent-filled compounds was calculated from crystallographic data of **2**, **4** and **5**, in which all uncoordinated solvent molecules inside the pores were deleted. The accessible pore volume was determined using the Mercury Pore Analyzer tool[1]. Based on this value, the solvent mass required to occupy the pore space in the solvated MOFs was estimated. This solvent contribution was added to the mass of the empty framework per unit cell, allowing the total densities of the three compounds in their fully solvated state to be calculated.

Table S2. Calculated densities for **2**, **4**, and **5**.

	Compound **2**	Compound **4**	Compound **5**
Network-accessible geometric volume* [cm^3]	$9.482\ 10^{-21}$	5.986E-21	6.293E-21
Framework density* [g/cm^3]	8.000E-01	6.470E-01	6.490E-01
Molar mass of framework [g/mol]	6.703E+03	3.119E+03	3.295E+03
Unit cell volume [cm^3]	1.391E-20	8.009E-21	8.430E-21
Solvent density [g/cm^3]	9.940E-01	7.860E-01	9.940E-01
Mass of void-occupying solvent per unit cell [g]	9.426E-21	4.705E-21	6.255E-21
Mass of empty framework per unit cell [g]	1.113E-20	5.180E-21	5.472E-21
Mass of pore-filled framework per unit cell [g]	2.056E-20	9.885E-21	1.173E-20
Total density [g/cm^3]	**1.478**	**1.234**	**1.391**

* calculated from Mercury Pore Analyzer software[1]

10. IR and Raman spectra of H_2dttc and 1

ATR-FTIR spectra of organic H_2dttc linker and MOFs were measured on a BRUKER VERTEX 70 with a SPECAC Golden Gate diamond ATR setup. Spectra were collected from 4000 to 400 cm^{-1} at 2 cm^{-1} resolution with 128 co-added scans (background recorded under identical conditions). MOF samples were handled in an Ar-filled glovebox.

Raman measurements of the organic H_2dttc linker and MOFs were performed with a Confocal Raman Microscope (CRS+) MonoVista from Spectroscopy & Imaging GmbH, using lasers with wavelengths of 514 nm and 785 nm. Samples of **1** were measured under inert conditions (Ar atmosphere) in quartz glass fluorescence cuvettes (1 cm path length).

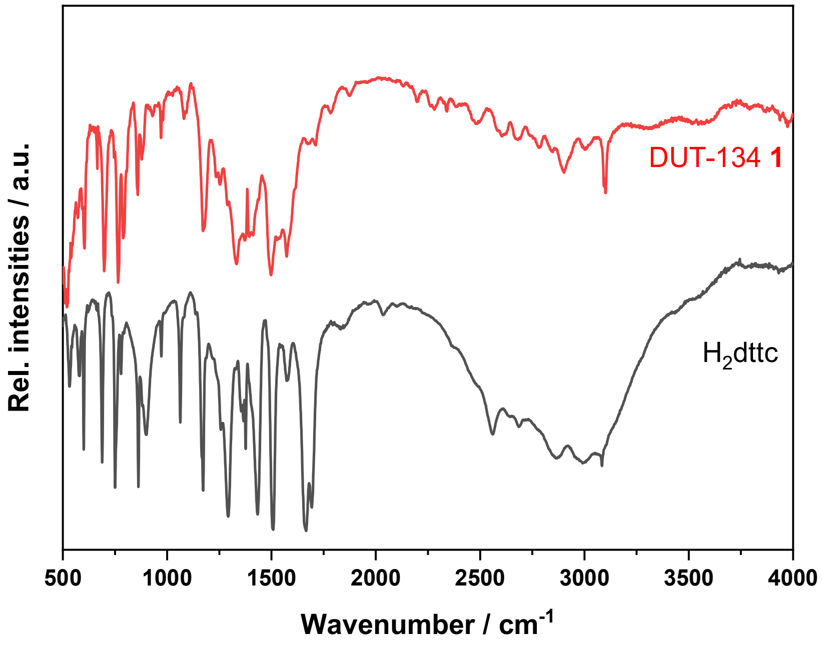

Figure S12. IR spectra of H_2dttc (black) and desolvated DUT-134 (compound **1**) (red).

In the spectra of H_2dttc, the bands at 1600 – 1800 cm^{-1} can be clearly assigned to the vibrations of the carboxylate groups, which correspond to the weak symmetrical valence band at 1632 cm^{-1} (Raman) and the strong IR-active asymmetric band at 1660 cm^{-1} (Figure S12, S13)[4]. The two most characteristic bands in the Raman spectrum at 1435 cm^{-1} and 1492 cm^{-1} are attributed to the dithienothiophene skeleton. The former results from collective vibrations of the entire alternating sequence of C=C/C-C bonds.

The weak Raman band at 1292 cm^{-1} and the easily observable intensive IR band at 1290 cm^{-1} show contributions from C-O stretching and in-plane C-OH bending modes[5]. In aromatic systems, C-H in-plane bending is typically observed within the range of 1000 – 1300 cm^{-1}, while C-H out-of-plane bending occurs in the 750 – 1000 cm^{-1} range. As stated by Socrates *et al.* [4], the in-plane bending of the linker H_2dttc is particularly evident in the IR spectrum, with a range of 1173 cm^{-1}

to 862 cm^{-1}. In contrast, the out-of-plane bending (γC-COOH, C-H) at 747 cm^{-1} can be identified through both Raman and IR spectroscopy.

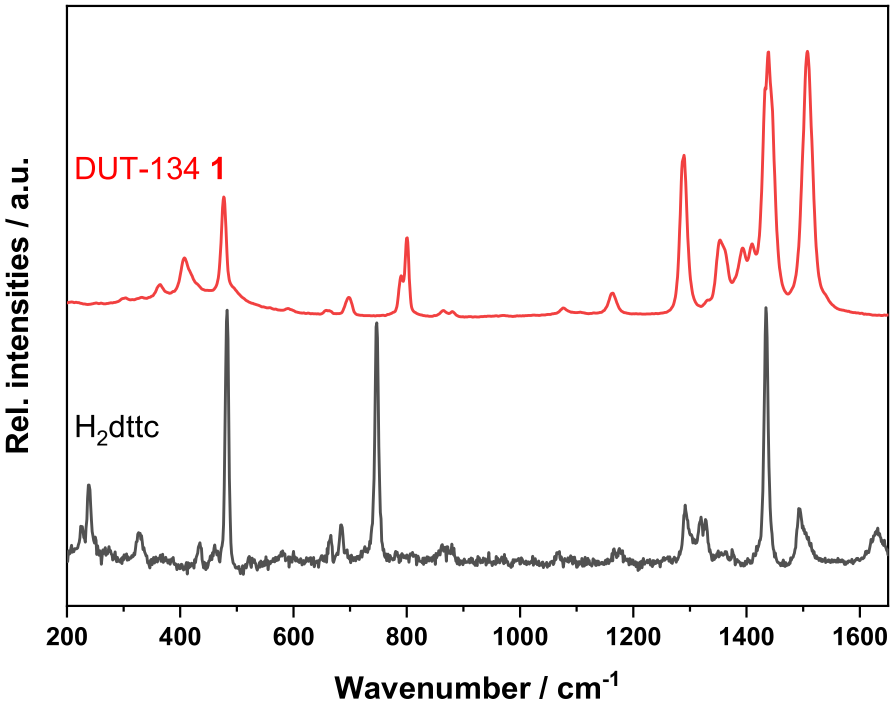

Figure S13. Raman spectra of H_2dttc (black) and desolvated DUT-134 (**1**) (red).

A comparison of the IR and Raman spectra of DUT-134 with those of the free linker H_2dttc reveals clear similarities in the vibrational motifs. In particular, the characteristic Raman bands of the dithienothiophene backbone (Line A and Line B) are preserved in the MOF structure at 1504 cm^{-1} and 1437 cm^{-1}. A straightforward assignment of MOF vibrational bands based solely on tabulated data of related compounds or by direct comparison with the linker spectra is, however, highly challenging and rarely reliable. While theoretical simulations can support such assignments, isotopic labelling experiments provide a far more precise and experimentally reliable method, offering the most accurate insight into the MOF vibrational modes.

To identify coordination-relevant vibrations, the linker was isotopically labelled at the carboxyl group with ^{13}C and ^{18}O. This approach enabled unambiguous identification of the two characteristic carboxylate stretching vibrations and the metal-oxygen vibrations of the MOF. The Raman spectra of isotopically labelled, desolvated DUT-134-^{13}C and DUT-134-^{18}O are directly compared with those of the unlabelled desolvated compound DUT-134 (**1**), as shown in

Figure S14. The bands at 1521 cm^{-1} and 1419 cm^{-1} were presumably assigned to $\nu_{as}(COO^-)$ and $\nu_s(COO^-)$, respectively. The isotopic substitution confirmed these assignments, yielding shifts of approximately 50 cm^{-1} and 35 cm^{-1} for DUT-134-^{13}C (1472 $cm^{-1}$and 1380 cm^{-1}) as well as 40 cm^{-1} and 33 cm^{-1} for DUT-134-^{18}O (1483 cm^{-1} and 1385 cm^{-1}; Figure S15). Interestingly, the expected stronger shifts of $\nu_{as}(COO^-)$ and $\nu_s(COO^-)$ for DUT-134-^{18}O compared to DUT-134-^{13}C, based on the higher mass change, were not observed. Instead, both isotopes produced shifts of similar magnitude, likely due to the overlap of vibrational modes leading to either reinforcement or attenuation of the shifts. The observed splitting of $\Delta\nu$ = 102 cm^{-1} between the asymmetric and symmetric carboxylate stretching vibrations is markedly smaller than the 160–180 cm^{-1} typically reported for paddle wheel type structures[4]. Nevertheless, isotopic 18abelling is considered a more specific and sensitive approach than those used in the literature, and the resulting assignments are regarded as reliable despite this deviation.

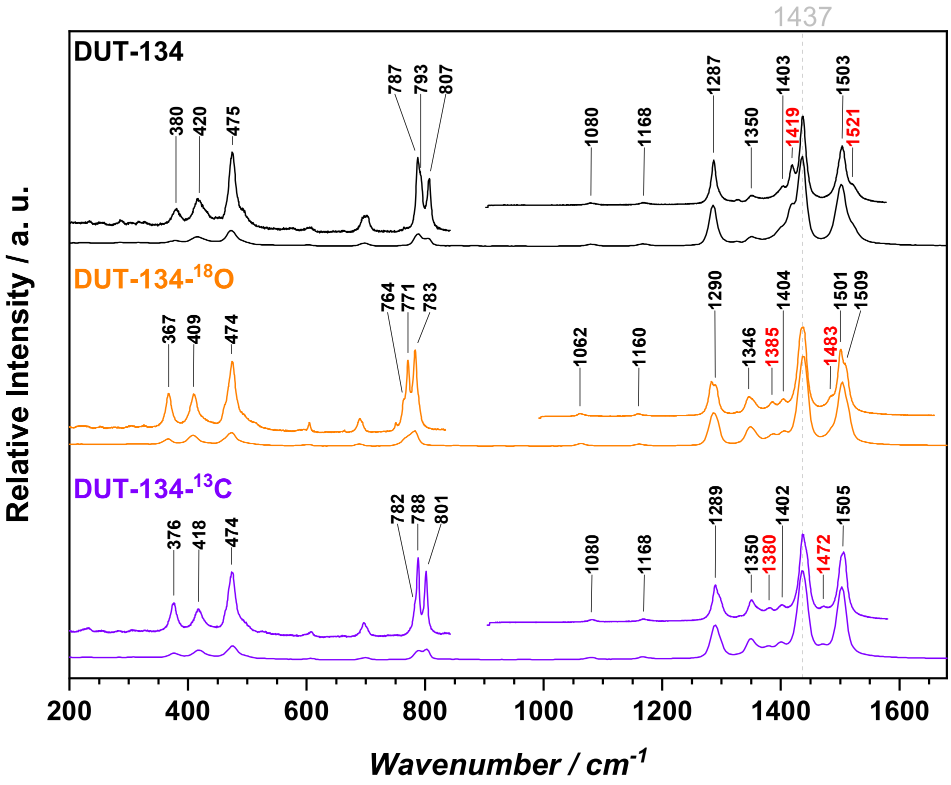

Figure S14. Raman spectra of **1** (black), DUT-134-^{18}O (orange), and DUT-134-^{13}C (purple); 514 nm.

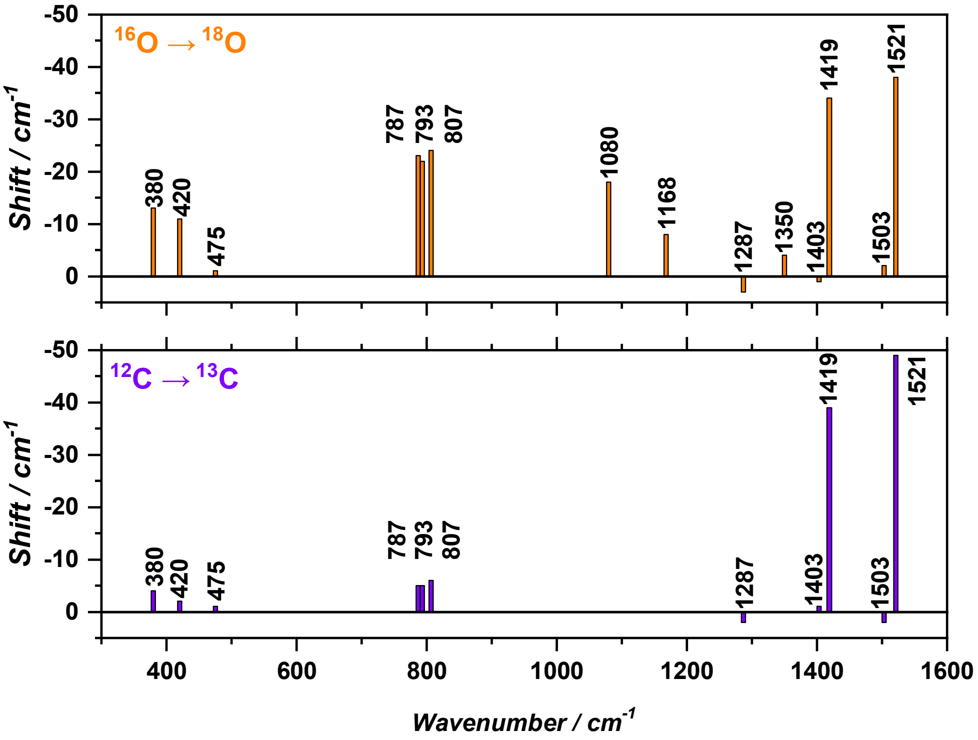

Figure S15. Effects of isotopic labeling in DUT-134: ^{18}O-labeling (orange) and ^{13}C-labeling (purple). The x-axis corresponds to the wavenumbers of the unlabeled MOF, while the bar height indicates the respective shifts of the vibrational bands.

Additional oxygen involvement is evident from bands at 1080 cm^{-1} and 1168 cm^{-1}, where ^{18}O-substitution induces significant shifts, whereas no changes are observed for ^{13}C. These bands could therefore be attributed to coupled in-plane bending vibrations of the carboxylate group. Furthermore, the out-of-plane bending vibration γ(C–COOH, C–H) around 800 cm^{-1} was confirmed, showing apparent shifts of more than 20 cm^{-1} for DUT-134-^{18}O and slightly smaller, but consistent, shifts for DUT-134-^{13}C.

In contrast, the band at ≈ 480 cm^{-1} remained unaffected by isotopic substitution, corroborating its assignment to aromatic ring vibrations and excluding any direct involvement in metal coordination. Cu–O vibrations were unambiguously identified to appear at 380 cm^{-1} and 420 cm^{-1} by ^{18}O-substitution, with additional smaller shifts observed for ^{13}C due to partial participation of the carbonyl carbon atom. All assignments discussed above were made for solvent-free DUT-134 (**1**). Upon coordination of DMF at the axial positions of the Cu PW units, pronounced spectral changes were observed. The DMF molecules introduce additional vibrational modes, most notably C=O stretching vibrations (1650 – 1700 cm^{-1}) and C–H deformation vibrations (1200 – 1450 cm^{-1}), which overlap with the carboxylate stretches of the MOF (Figure S16)[4].

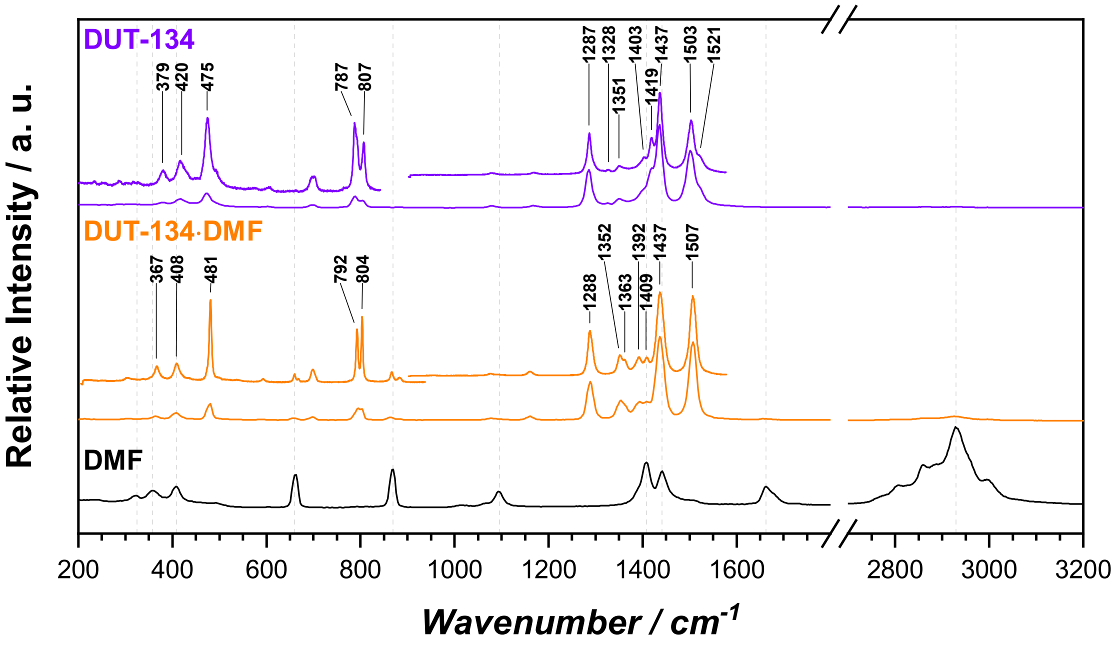

Figure S16. Raman spectra of **1** (purple), **2** (orange), and DMF (black); 514 nm.

Consequently, $\nu_{as}(COO^-)$ at 1521 cm^{-1} is no longer clearly resolved in **2** and appears overlapped by an intense band at 1507 cm^{-1} ($\Delta\nu \approx 14$ cm^{-1}). For $\nu_s(COO^-)$, a shift of similar magnitude from 1419 cm^{-1} to 1409 cm^{-1} can be observed. At lower wavenumbers, the Cu–O stretching modes observed at 420 cm^{-1} and 380 cm^{-1} in **1** shift to 410 cm^{-1} and 370 cm^{-1}, respectively. This redshift is consistent with a slight weakening of the Cu–O bond upon DMF coordination, reflecting a reduction in the bond force constants caused by interaction with the polar solvent.

Even with isotopic 20abelling, the COO^- stretching vibrations of **2** cannot be assigned unambiguously. In both **3a** and **3b**, the asymmetric stretching mode is not clearly distinguishable and is likely overlapped by the intense band at 1437 cm^{-1}. The $\nu_s(COO^-)$ stretching modes can be identified only in **2** and **3b** at 1410 cm^{-1} and 1378 cm^{-1}, respectively, both showing a redshift of ≈10 cm^{-1} compared to the solvent-free structures. In contrast, the out-of-plane bending modes around 800 cm^{-1} remain well resolved and exhibit the expected isotopic shifts ($\Delta\nu_{^{13}C} \approx 6$ cm^{-1}; $\Delta\nu_{^{18}O} \approx 25$ cm^{-1}). The low-frequency Raman bands at 367 cm^{-1} and 408 cm^{-1} are similarly affected, with shifts comparable to those observed in solvent-free DUT-134 ($\Delta\nu_{^{13}C} < 5$ cm^{-1}; $\Delta\nu_{^{18}O} \approx 10$–15 cm^{-1}). These findings confirm the reliability of isotopic 20abelling as a method for vibrational band assignment, even in the presence of DMF coordination.

11. Theoretical IR and Raman spectra of solvent-free DUT-134

The calculations of IR and Raman spectra were carried out as described in the "Methods and Instrumentation" section of the main manuscript. They are in good agreement with the experimentally acquired data (Figure S17).

Table S3. Comparison of the peak positions of the calculated and experimental Raman spectra of desolvated DUT-134 **1**. Note that Raman activity was not evaluated in method (i).

**Peak position Procedure I / cm^{-1}**	**Peak position Procedure II / cm^{-1}**	**Experimental data / cm^{-1}**
368, 370, 372, 373	365	375
415 – 422	421	419
458 – 468, 470, 471, 475 – 482	458	473
521 – 523, 532-537	525	526
593 – 602	600	607
691 – 696	692	699
730 – 735	734	790
782	788	805
1061 – 1066, 1170 – 1071, 1073, 1080 – 1081	1063	1076
1151 – 1161,	1155	1164
1275 – 1276,	1272	1285
1334, 1336 – 1341	1330	1325
1345, 1347, 1349	1347	1348
1382 – 1385, 1389 – 1395, 1400 – 1405	1384	1399
1436 – 1440	1439	1419
1457 – 1478	1451	1436
1480 – 1481	1480	1501
1483 – 1491, 1535 – 1539	1489	1515

a)

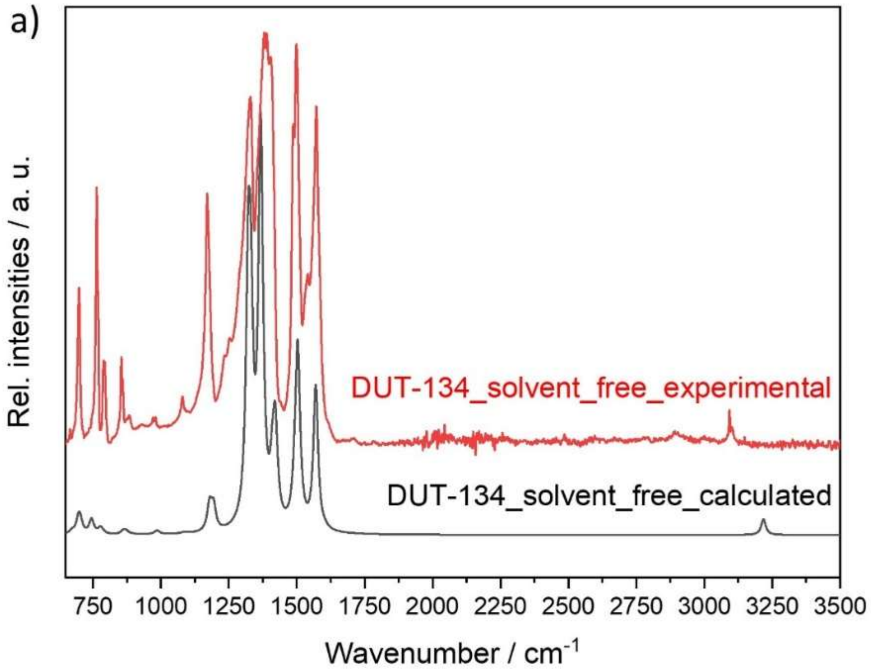
DUT-134_solvent_free_experimental
DUT-134_solvent_free_calculated
Rel. intensities / a. u.
750 1000 1250 1500 1750 2000 2250 2500 2750 3000 3250 3500
Wavenumber / cm-1

b)

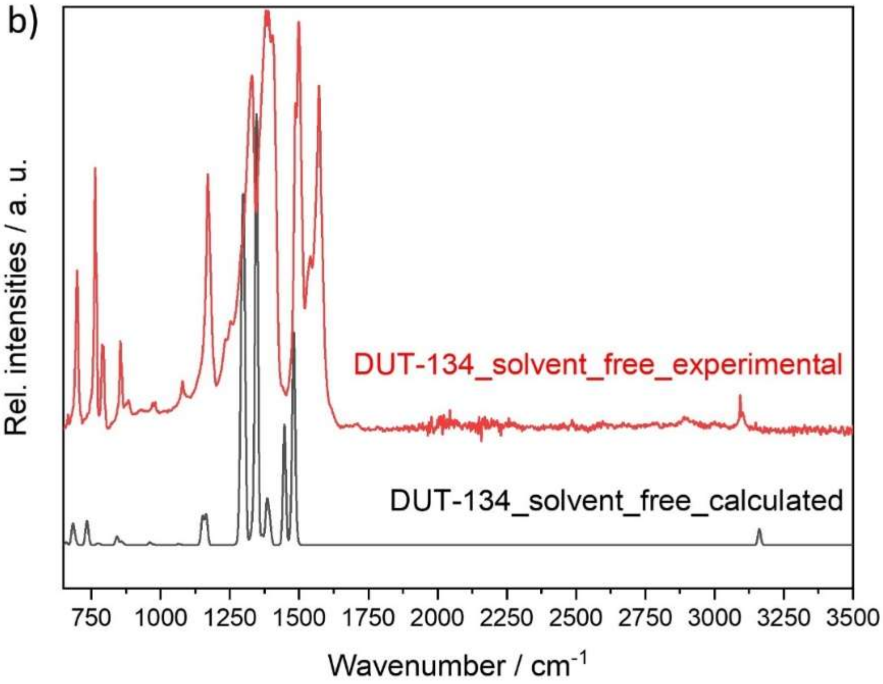
DUT-134_solvent_free_experimental
DUT-134_solvent_free_calculated
Rel. intensities / a. u.
750 1000 1250 1500 1750 2000 2250 2500 2750 3000 3250 3500
Wavenumber / cm-1

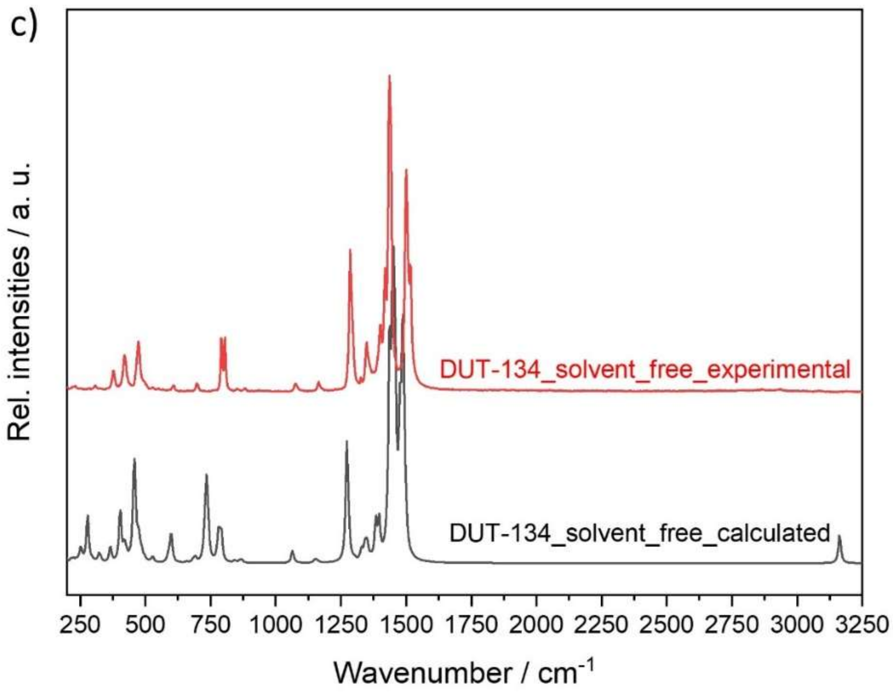

Figure S17. a) Calculated (procedure i) (black) and experimental (red) IR spectra of solvent-free DUT-134 (**1**); b) Calculated (procedure ii) (black) and experimental (red) IR spectra of solvent-free DUT-134 (**1**); c) Calculated (procedure ii) (black) and experimental (red) Raman spectra of solvent-free DUT-134 (**1**).

12. Raman spectra of 2, 4, 5, 7 and 8

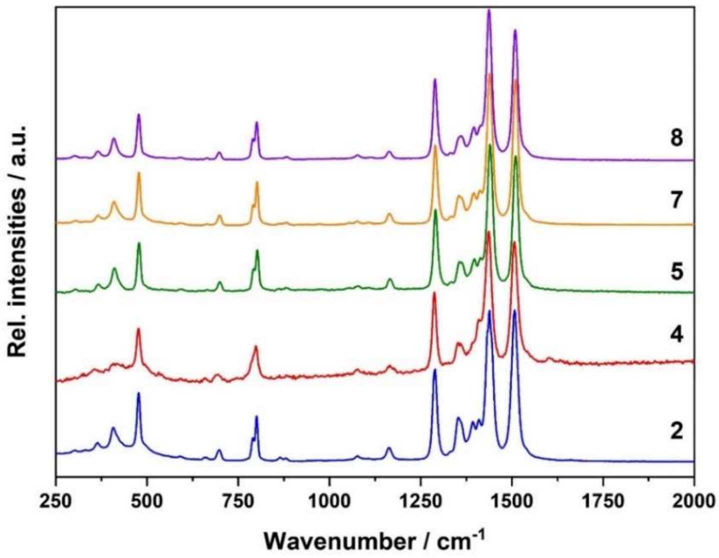

Figure S18. Experimental Raman spectra of **2** (blue), **4** (red), **5** (green), **7** (orange), and **8** (purple) collected with a laser wavelength of 532nm and a beam diameter of 2.1 µm.

13. Scanning Electron Microscopy (SEM)

Scanning electron microscopy (SEM) measurements of **1** were carried out using a 2 kV acceleration voltage and a working distance of 12 mm on a SU8020 from Hitachi. Prior to the measurements, the samples were sputtered with gold to increase the conductivity.

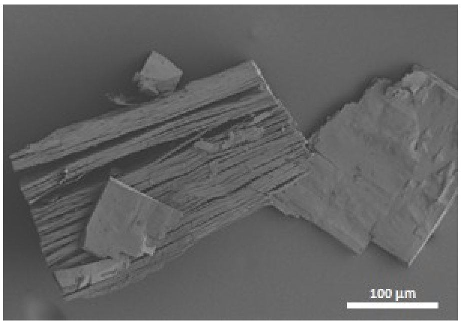

Figure S19. SEM image of crystals of **1** after desolvation employing supercritical CO_2.

14. Pawley refinement of compounds 5, 7 and 8

Crystals of **5** were analyzed by powder X-ray diffraction. The indexing resulted in a unit cell, similar to that of **4**. The unit cell of **4** was used as an initial model for the Pawley fit, which shows a good convergence between the calculated and theoretical profiles (Fig. S20).

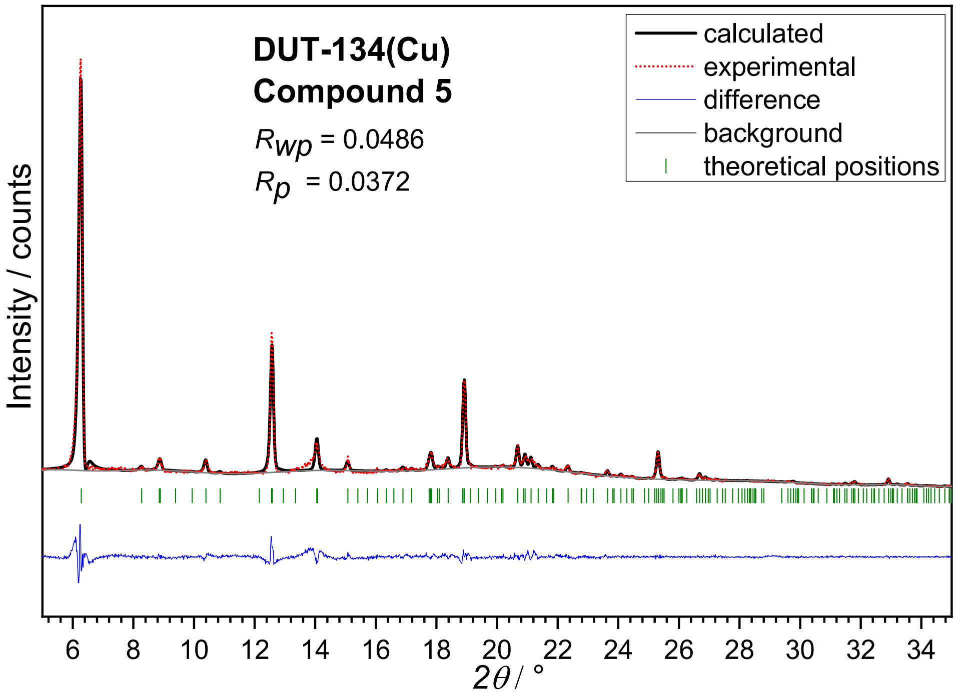

Figure S20. Pawley fit for powder X-ray diffraction pattern of **5**.

Table S4. Crystallographic data for DUT-134_DMF (**5**).

	DUT-134_DMF (**5**)
Crystal system, space group	Tetragonal, *P4/nmm* (no. 129)
Unit cell dimensions [Å]	*a* = 28.113(1); *b* = 28.113(1); *c* = 10.666(3)
Cell volume [Å^3]	8430(1)

Crystals of **7** and **8** were analyzed by powder X-ray diffraction. The indexing resulted in a unit cell, similar to that of **2**. The unit cell of **2** was used as an initial model for the Pawley fit, which shows a good convergence between the calculated and theoretical profiles (Fig. S21,S22).

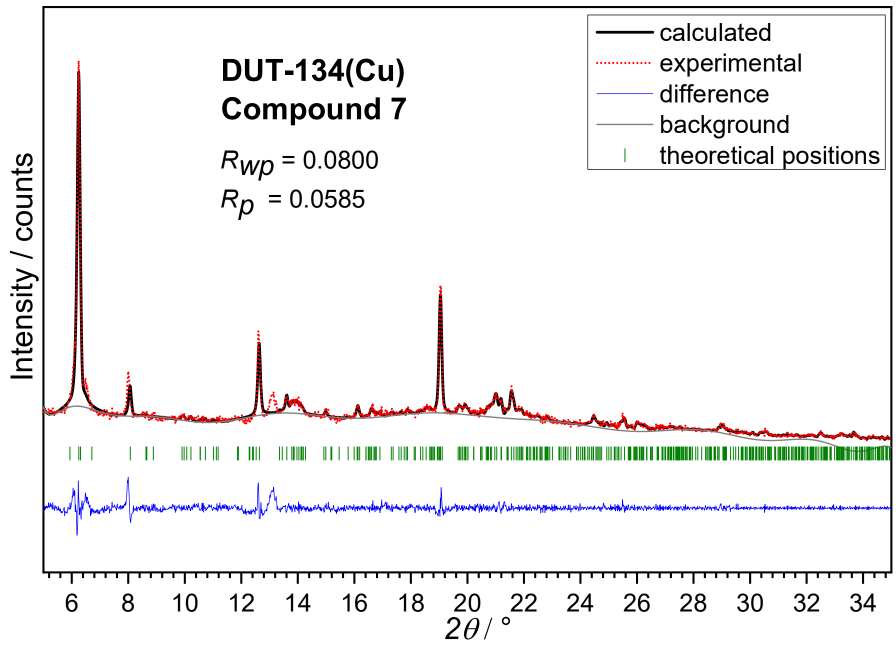

Figure S21. Pawley fit for powder X-ray diffraction pattern of **7**.

Table S5. Crystallographic data for DUT-134_Pentanitrile (**7**).

	DUT-134_Pentanitrile (**7**)
Crystal system, space group	Orthorhombic, *Pnma* (no. 62)
Unit cell dimensions [Å]	*a* = 17.551(9); *b* = 28.217(7); *c* = 27.978(7)
Volume [Å^3]	13857(1)

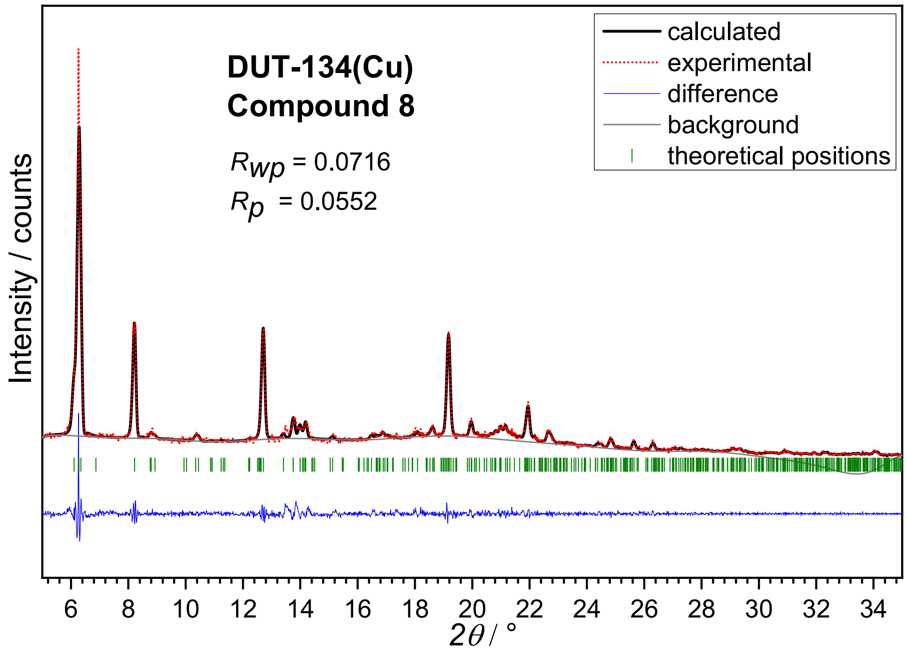

Figure S22. Pawley fit for powder X-ray diffraction pattern of **8**.

Table S6. Crystallographic data for DUT-134_Heptanitrile (**8**).

	DUT-134_Heptanitrile (**8**)
Crystal system, space group	Orthorhombic, *Pnma* (no. 62)
Unit cell dimensions [Å]	*a* = 16.912(6); *b* = 28.133(8); *c* = 27.824(8)
Volume [Å^3]	13239(5)

15. Analysis of *in situ* PXRD amorphous background for dead time determination

In order to determine the dead time of the experimental flow cell setup for in situ powder X-ray diffraction, the amorphous background was subtracted from the experimental data (Fig. S23, S24). Subsequently, for each frame (i.e., each diffractogram measured), the data were fitted to a polynomial function. Finally, the second derivatives of each function were compared to the previous one to detect slight changes in the overall amorphous background integral more clearly. The differences were subsequently plotted as a function of measurement time to accurately estimate the solvent arrival time at the sample (Fig. S25, S26).

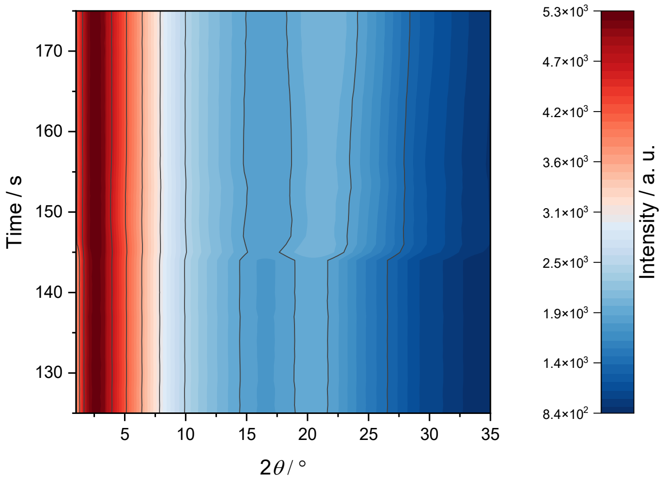

Figure S23. Extracted amorphous background diffractogram heatmap from *in situ* powder X-ray diffractograms of ligand exchange from **2** to **4**.

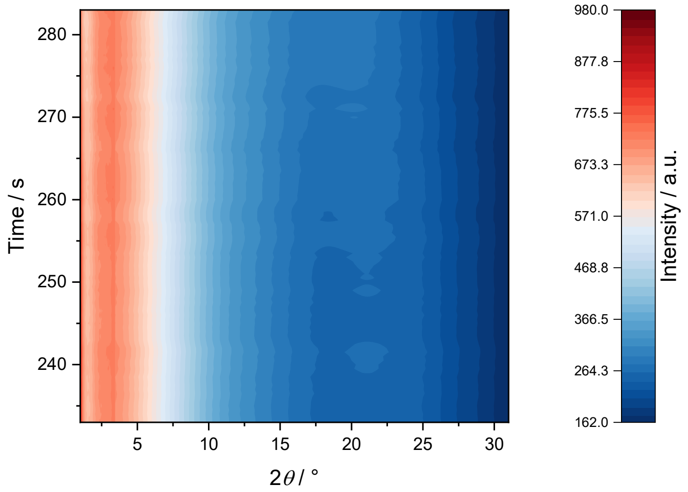

Figure S24. Extracted amorphous background diffractogram heatmap from *in situ* powder X-ray diffractograms of ligand exchange from **4** to **5**.

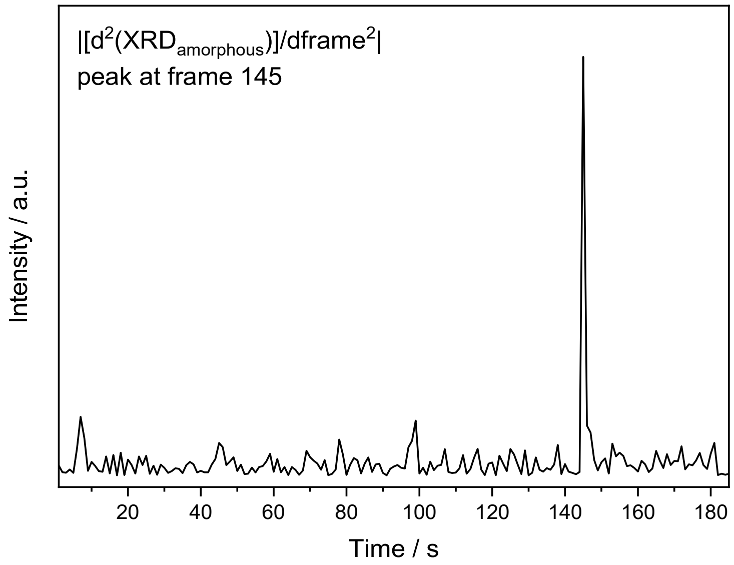

Figure S25. Second derivative change for phase transition **2** to **4** with peak at frame 145 (i.e., after 145 seconds).

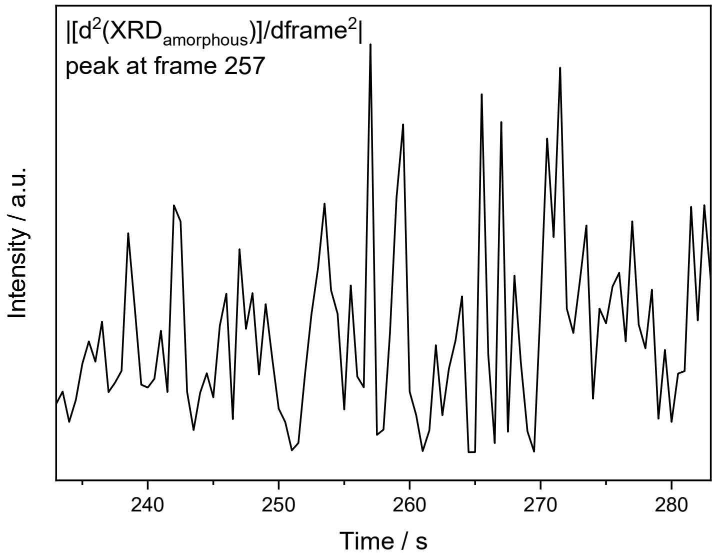

Figure S26. Second derivative change for phase transition **4** to **5** showing peak at frame 257.

16. Script for analysis of PXRD amorphous background

The amorphous background of the conducted *in situ* powder X-ray diffractograms was analyzed through a Python script that employed the 'peakutils.baseline' fitting algorithm. The script in its entirety is presented in a Zenodo repository[6].

17. Cu-O Raman band shift upon solvent exchange

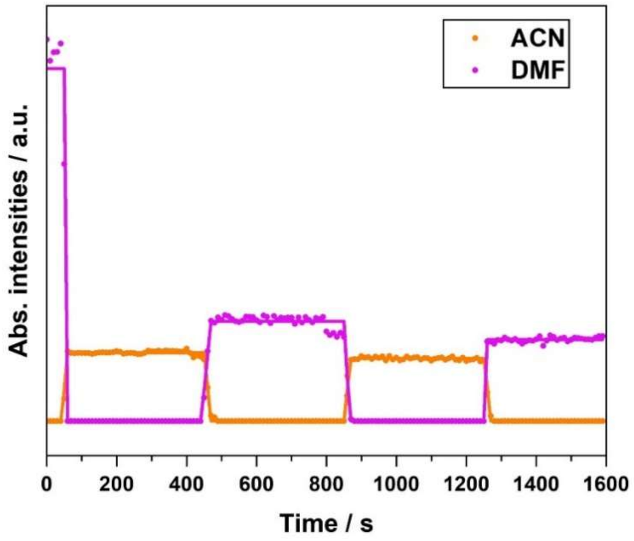

Figure S27. Absolute intensities of the bands at 860 cm^{-1} (DMF, purple) and at 2251 cm^{-1} (ACN, orange) as a function of time during solvent exchange.

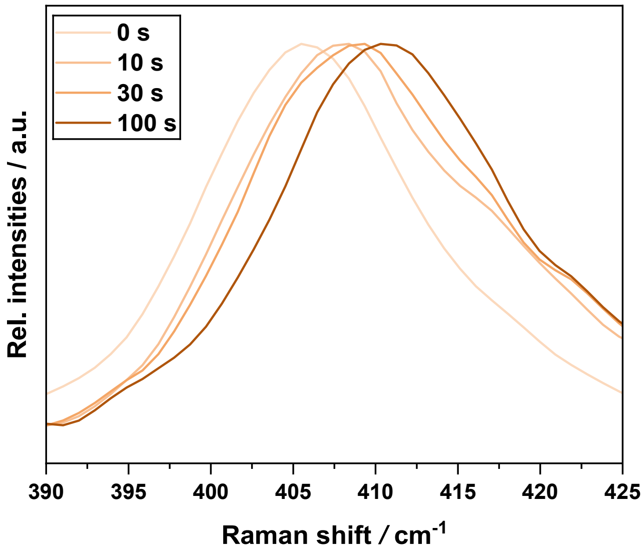

Figure S28. Raman spectra of the Cu-O stretching band during the solvent exchange from DMF **2** to pentanenitrile **7** (from light orange at 0 seconds after dead time to dark orange at 100 seconds after dead time).

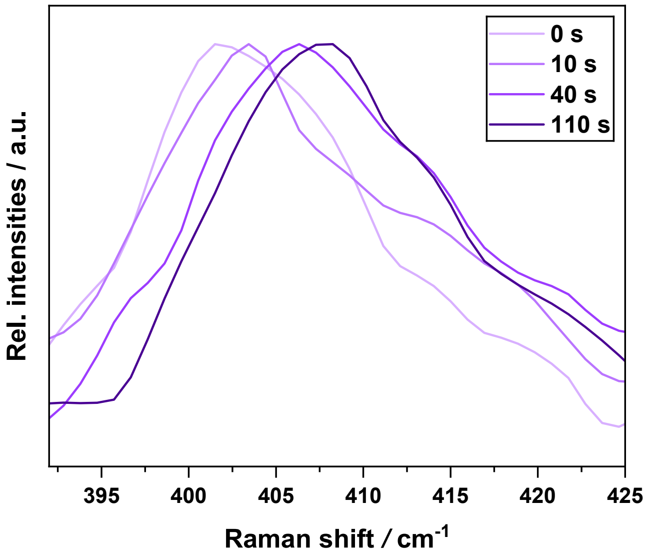

Figure S29. Raman spectra of the Cu-O stretching band during the solvent exchange from DMF **2** to heptanenitrile **8** (from light purple at 0 seconds after dead time to dark purple at 110 seconds after dead time).

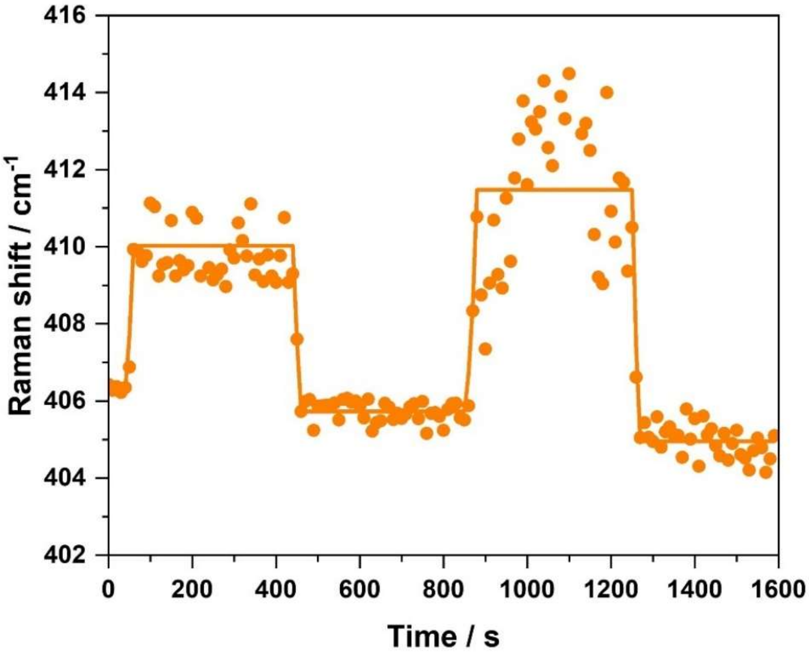

Figure S30. Wavenumber of the Cu-O stretching band during the cycling solvent exchange of DMF and pentanenitrile.

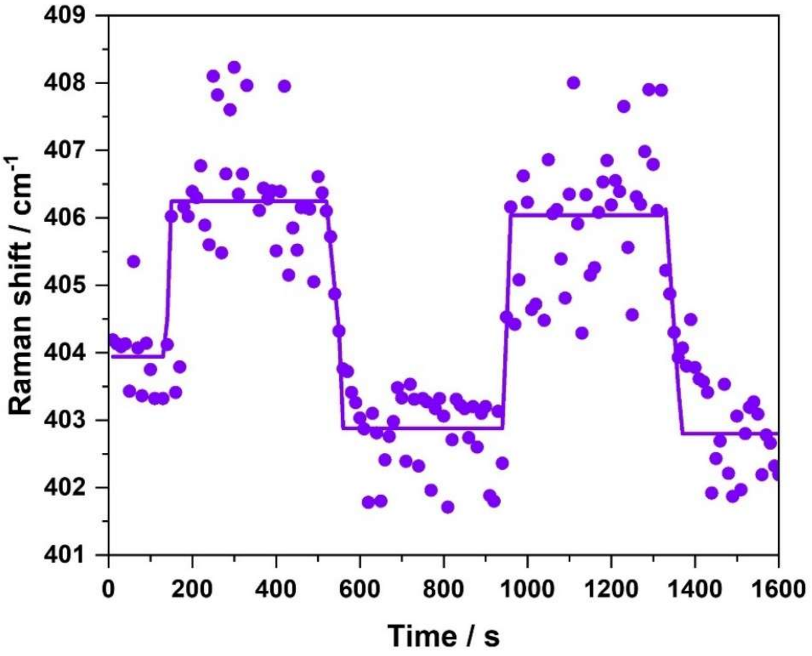

Figure S31. Wavenumber of the Cu-O stretching Raman band during the cycling solvent exchange of DMF and heptanenitrile.

18. Ligand exchange kinetics for acetonitrile, heptanenitrile, and pentanenitrile

The ligand exchange kinetics for DMF with acetonitrile, heptanenitrile and pentanenitrile were calculated from *in situ* Raman data with linear fit curves.

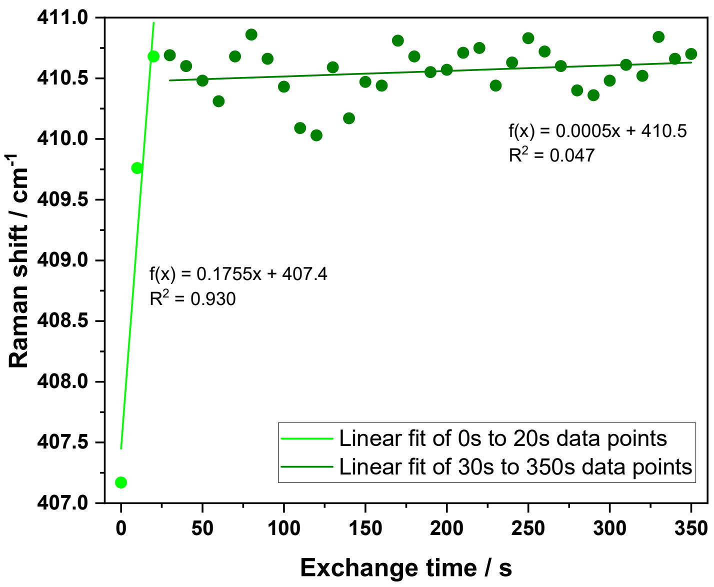

Figure S32. Wavenumber of the Cu-O stretching Raman band during solvent exchange of DMF to can (**2** to **4**), with linear fits from 0 s to 20 s of experiment time and 30 s to 350 s.

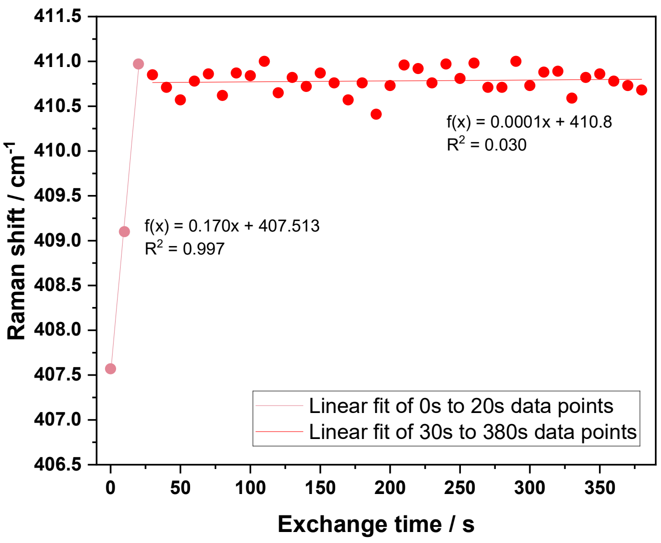

Figure S33. Wavenumber of Cu-O stretching Raman band during solvent exchange of DMF to ACN after one cycle (**5** to **4**), with linear fits from 0 s to 20 s of experiment time and 30 s to 380 s.

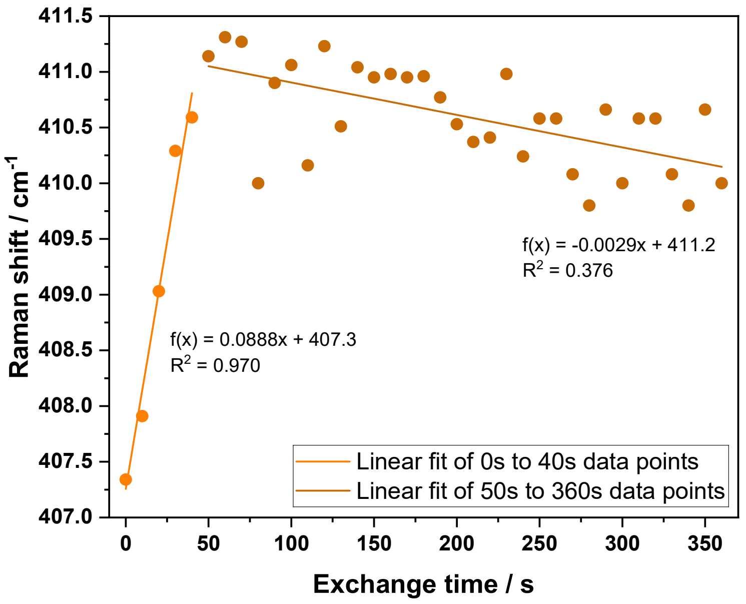

Figure S34. Wavenumber of the Cu-O stretching Raman band during solvent exchange of DMF to pentanenitrile (**2** to **7**), with linear fits from 0 s to 40 s of experiment time and 50 s to 360 s.

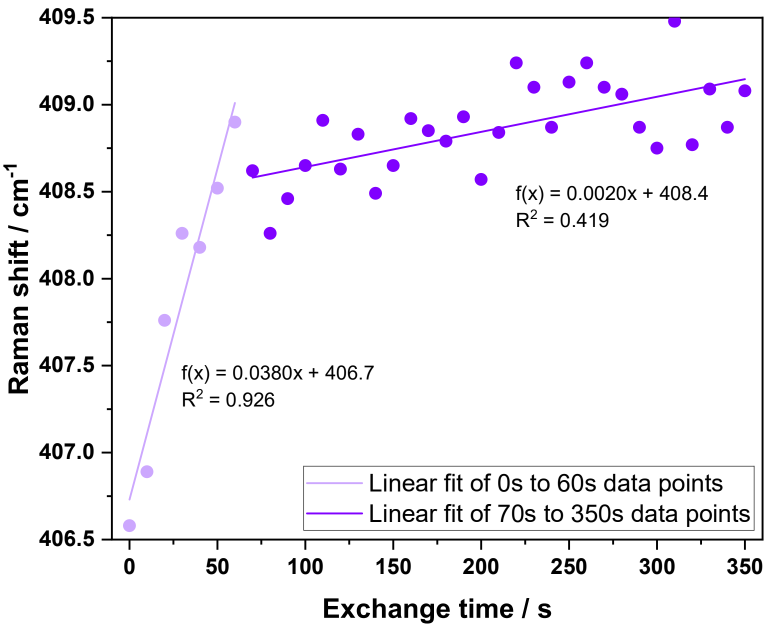

Figure S35. Wavenumber of the Cu-O stretching Raman band during solvent exchange of DMF to heptanenitrile (**2** to **8**), with linear fits from 0 s to 60 s of experiment time and 70 s to 350 s.

References

(1) Macrae, C. F.; Sovago, I.; Cottrell, S. J.; Galek, P. T. A.; McCabe, P.; Pidcock, E.; Platings, M.; Shields, G. P.; Stevens, J. S.; Towler, M.; Wood, P. A. Mercury 4.0: from visualization to analysis, design and prediction. *J. Appl. Crystallogr.* **2020**, *53*, 226–235. DOI: 10.1107/S1600576719014092.

(2) Lucas Hermann Negri; Christophe Vestri. *lucashn/peakutils: v1.1.0*; Zenodo, 2017.

(3) Schwotzer, F.; Senkovska, I.; Bon, V.; Lochmann, S.; Evans, J. D.; Pohl, D.; Rellinghaus, B.; Kaskel, S. Solvent-assisted delamination of layered copper dithienothiophene-dicarboxylate (DUT-134). *Inorg. Chem. Front.* **2021**, *8*, 3308–3316. DOI: 10.1039/D1QI00349F.

(4) Socrates, G. *Infrared and Raman characteristic group frequencies: tables and charts*; John Wiley & Sons, 2004.

(5) Magdaline, J. D.; Chithambarathanu, T. Vibrational spectra (FT-IR, FT-Raman), NBO and HOMO, LUMO studies of 2-thiophene carboxylic acid based on density functional method. *IOSR J. Appl. Chem.* **2015**, *8*, 6–14. DOI: 10.9790/5736-08510614.

(6) Engemann, R.; Senkovska, I.; Schwotzer, F.; Winkler, J.; Bon, V.; Machill, S.; Wollmann, P.; Reichmayr, F.; Weiß, J.; Scheffler, J.; Bas, E. E.; Formalik, F.; Snurr, R. Q.; Golze, D.; Weidinger, I. M.; Brunner, E.; Kaskel, S. *Data Publication for in situ insights into 2D MOF solvent-assisted restacking and active site exchange kinetics*; Zenodo, 2025. DOI: 10.5281/zenodo.16901296.
